# SLC26A11 Inhibition Reduces Oncotic Neuronal Death and Attenuates Stroke Reperfusion Injury

**DOI:** 10.1007/s12035-023-03453-1

**Published:** 2023-06-28

**Authors:** Shunhui Wei, Bo Chen, See Wee Low, Charlene Priscilla Poore, Yahui Gao, Bernd Nilius, Ping Liao

**Affiliations:** 1 Calcium Signalling Laboratory, Department of Research, National Neuroscience Institute, 11 Jalan Tan Tock Seng, Singapore, 308433 Singapore; 2 Present Address: Department of Chemical and Biomolecular Engineering, National University of Singapore, Singapore, 119077 Singapore; 3 Department of Cellular and Molecular Medicine, KU Leuven, 3000 Louvain, Belgium; 4 Duke-NUS Medical School, Singapore, 169857 Singapore; 5 Health and Social Sciences, Singapore Institute of Technology, Singapore, 138683 Singapore

**Keywords:** Ischemic stroke, SLC26A11, Swelling, Cell death, Reperfusion injury

## Abstract

Neuronal swelling is a pathological feature of stroke which contributes to the formation of cytotoxic edema. Under hypoxic condition, aberrant accumulation of sodium and chloride ions inside neurons increases osmotic pressure, leading to cell volume increase. Sodium entry pathway in neurons has been studied extensively. Here, we determine whether SLC26A11 is the major chloride entry pathway under hypoxia and could be the target for protection against ischemic stroke. In this study, electrophysiological properties of chloride current in primary cultured neurons were characterized using low chloride solution, 4,4′-diisothiocyano-2,2′-stilbenedisulfonic acid, and SLC26A11-specific siRNA under physiological conditions or ATP-depleted conditions. In vivo effect of SLC26A11 was evaluated on a rat stroke reperfusion model. We found that SLC26A11 mRNA in primary cultured neurons was upregulated as early as 6 h after oxygen glucose deprivation, and later, the protein level was elevated accordingly. Blockade of SLC26A11 activity could reduce chloride entry and attenuate hypoxia-induced neuronal swelling. In the animal stroke model, SLC26A11 upregulation was mainly located in surviving neurons close to the infarct core. SLC26A11 inhibition ameliorates infarct formation and improves functional recovery. These findings demonstrate that SLC26A11 is a major pathway for chloride entry in stroke, contributing to neuronal swelling. Inhibition of SLC26A11 could be a novel therapeutic strategy for stroke.

**Supplementary Information:**

The online version contains supplementary material available at 10.1007/s12035-023-03453-1.

## Introduction

Oncotic cell death or oncosis is a major type of cell death following stroke. The most prominent feature of oncosis is cellular swelling which is caused by hypoxia/ischemia [1]. After stroke, energy depletion leads to the failure of ATP-dependent pumps that are important to maintain membrane potential. An increased membrane permeability facilitates transmembrane ionic movement down their respective concentration gradients. Sodium, calcium, and chloride ions flux into cell and potassium ions flow outside cell. Intracellular space also contains abundant negatively charged proteins; however, their relatively large size limits the transmembrane movement. When ionic influx exceeds efflux, a higher intracellular osmotic pressure is established, leading to water influx and subsequent oncosis [1].

As extracellular Na^+^ concentration (140–145 mM) is much higher than Ca^2+^ (1.8 mM) [1], Na^+^ entry is likely to contribute more to increase intracellular osmotic pressure than Ca^2+^. Currently, multiple Na^+^ entry pathways have been identified during the development of oncosis in stroke. These include glutamate receptors [2], acid-sensing ion channels (ASICs) [3], transient receptor potential channels in particular TRPM4 [4], TRPV5 [5], and various exchangers [1]. Interestingly, activation of voltage-sensitive sodium channels (VSSC) tends to induce apoptotic neuronal death during oxygen deprivation [6]. To achieve electroneutrality, Na^+^ entry needs to be balanced by an influx of negatively charged ions, in particular Cl^−^. One pathway for Cl^−^ influx is Na^+^-K^+^-2Cl^−^ co-transporter 1 (NKCC1) [7]. However, due to the stoichiometry of the transported ions (1 Na^+^: 1 K^+^: 2 Cl^−^) from NKCC1, and its efficiency and expression level, Cl^−^ entry via NKCC1 is not sufficient to balance the Na^+^ influx via multiple pathways. There may exist additional pathway conducting Cl^−^ currents during stroke.

Recently, SLC26A11 has been identified as a novel voltage-gated chloride channel during excitotoxic swelling [8]. Blocking SLC26A11 could attenuate neuronal oncosis induced by Na^+^ influx via sustained activation of VSSC or glutamate receptors. In this study, we examined the role of SLC26A11 in cortical neurons during hypoxia. The in vivo effect of blocking SLC26A11 under hypoxic conditions was further studied using a rat model of stroke reperfusion.

## Materials and Methods

### Animal Model and Study Design

This study was approved and conducted in accordance with the guidelines of the Institutional Animal Care and Use Committee of the National Neuroscience Institute, Singapore (IACUC Protocol No. A19102). The stroke model has been described previously [9]. In brief, male Sprague Dawley rats weighing approximately 250–280 g were anesthetized with ketamine (75 mg/kg) and xylazine (10 mg/kg) intraperitoneally. Rectal temperature was monitored using a rectal probe and maintained at 36.5–37.0 °C, connecting to the PowerLab 4/35 by a T-type pod (MLT1403&312, RET-2, AD Instruments). To create middle cerebral artery occlusion (MCAO), the left common carotid artery (CCA), internal carotid artery (ICA), and external carotid artery (ECA) were dissected out. A silicon-coated filament (0.37 mm, Cat #403756PK10, Doccol Corp, CA, USA) was introduced into the left ICA through ECA. Cerebral blood flow of the animals was monitored by a laser Doppler flowmetry (moorVMS-LDF2™, Moor Instruments Inc., DE, USA). Reperfusion was achieved by removing the filament gently from the ECA at 3 h following occlusion. Criteria for animal exclusion are as follows: rats with ≤ 70% cerebral blood flow reduction, rats without motor functional deficit assessed by rotarod test at 1 day following occlusion, and rats died during the observation periods. During the experiment, the animals were monitored daily. All researchers involved in the study were blinded to the intervention.

### Primary Culture of Rat Cortical Neurons

Primary culture of cortical neurons has been described previously [4]. In brief, fetal brains from embryonic day 18 (E18) pregnant Sprague Dawley rats with mixed sex were obtained and digested for 40 min in Earle’s balanced salt solution (EBSS, Thermo Fisher Scientific, MA, USA) containing 20-U/ml papain (Worthington, Lakewood, NJ, USA). Dissociated cells were seeded on 12-mm round glass coverslips coated with poly-L-lysine and laminin or placed into 60-mm tissue culture dishes with neuron culture medium containing Neurobasal medium, 2% B27 supplement, and 1% GlutaMAX supplement (Thermo Fisher Scientific, MA, USA). Medium was replaced 1 day after plating, and half of the medium was changed every 3 days. The cells were treated with 4-µM cytosine arabinoside from days in vitro (DIV) 3–6 to restrict mitotic cell proliferation and maintained for 10–21 days in neuron culture medium at 37 °C.

### ATP Depletion and In Vitro Hypoxic Induction

ATP depletion during patch clamp recording, the neurons were perfused with an anoxic artificial cerebrospinal fluid (aCSF) containing 5-mM NaN_3_ and 10-mM 2-deoxyglucose to reduce glycolytic capacity and interfere with cellular respiration in energy metabolism [4].

For 24-h OGD (oxygen–glucose deprivation), the cells were grown in hypoxic media and placed in a polycarbonate hypoxia induction chamber (Modular Incubator Chamber, #27,310, STEMCELL Technologies Inc., Vancouver, BC, Canada). Hypoxic chamber creates and maintains a hypoxic environment for cell culture without alternating cell behavior by chemicals. The chamber was first flushed with a gas mixture containing 1% O_2_, 5% CO_2_, and 94% N_2_ for 5 min to purge the ambient air from the chamber. Following that, the hypoxia chamber was tightly sealed and placed in a 37 °C incubator for 24 h. The hypoxic medium for neurons contains low glucose media (2 parts of EBSS mixed with 1 part of low glucose DMEM, Invitrogen, Life Technologies Corporation, USA).

### Immunofluorescence Staining and Western Blot

Immunofluorescence staining was performed as previously described [10]. In brief, the rats were sacrificed and perfused 1 day after stroke induction. Then, the brains were harvested and sectioned at 10 µm in thickness. Following fixation with 4% paraformaldehyde, the brain slice was incubated in 100-μl blocking serum (10% fetal bovine serum in 0.2% PBST) for 1 h. Primary antibodies include anti-SLC26A11 (HPA029893, Sigma-Aldrich, MO, USA, 1:100), anti-NeuN (MAB377, Millipore, MA, USA, 1:250), and anti-MAP2 (M4403, Sigma-Aldrich, MI, USA, 1:250 for samples from neuronal cultures). Secondary antibodies are conjugated with FITC or Alexa Fluor 594. Fluorescent images were captured with a laser scanning confocal microscope system (FV3000 with software FV31S-SW, Olympus, Tokyo, Japan). Olympus IX 83 inverted microscope with motorized XY stage and 40X Oil Objective, X-Apo, 1.40NA/0.13WD was used. Laser diodes: 405 nm (50mW); 488 nm (20mW); 561 nm (20mW); 640 nm (40mW). Detectors: FV31-SD 2 CH spectral detector unit by Multi-Alkali PMT, FV31-HSD 2CH High Sensitivity-Spectral detector unit by Cooled GaAsP PMT (Olympus, Tokyo, Japan).

For western blot of rat brains, tissues from ipsilateral and the corresponding contralateral hemispheres were harvested. Infarct area which was negatively stained by TTC was excluded. For cultured cells, the cells were grown in 60-mm culture dishes and harvested after hypoxia treatment to extract total protein. To perform western blot, 30 µg of total protein was resolved on 10% SDS-PAGE gels at 80 V and electrophoretically transferred to PVDF membranes (1,620,177, Bio-Rad, CA, USA) at 100 V for 2 h at 4 °C. After blocking with StartingBlock (PBS) blocking buffer (37,538, Thermo Fisher Scientific, MA, USA) for 1 h at room temperature, membranes were incubated overnight at 4 °C with primary antibodies: anti SLC26A11 (HPA029893, Sigma-Aldrich, MO, USA) and anti-actin (A1978, Sigma-Aldrich, MI, USA, 1:5000). After washing away primary antibodies, the membranes were incubated with secondary antibodies (A4416 or A4914, Sigma-Aldrich, MI, USA, 1:5000) for 1 h at room temperature. Amersham ECL Western Blotting Analysis System (RPN2109, GE Healthcare, IL, USA) was used, and the bands were visualized using medical X-ray processor (MXP-2000, KODAK, NY, USA). Quantification was performed using ImageJ.

### In Vitro and In Vivo siRNA Delivery

For cultured neurons, siRNA was transiently co-expressed with GFP using Lipofectamine 2000 reagent transfection method following its protocol (Thermo Fisher Scientific, MA 02451, USA). Twenty-four to forty-eight hours after transfection, the cells were fixed with 4% paraformaldehyde, followed by immunofluorescence staining. ImageJ was used to quantify the fluorescent intensity. For electrophysiological study, the GFP-positive cells were selected for patch clamping as successful transfection.

SLC26A11 siRNA has been described previously which could inhibit SLC26A11 expression [8]. The sense sequence is 5′-mGmCrAmUrGmUrCrArGrCrAmArUmArUmArGmArCrUrArCrAmCC-3′, and the antisense sequence is 5′-rGrGrUrGrUrArGrUrCrUrArUrArUrUrGrCrUrGrArCrArUrGrCrGrU-3′. The in vivo siRNA was purchased from IDT (Integrated DNA Technologies Pte. Ltd., Coralville, IA, USA). A single dose of 25-nmol siRNA was selected to be given intravenously immediately after occlusion. Control animals received a same dose of scrambled DsiRNA (negative control DsiRNA, Cat # 51–01-14–04, IDT, Coralville, IA, USA) after stroke induction.

### Electrophysiology

Whole-cell patch clamp was used to measure SLC26A11 currents in neurons grown in 24-well plates and co-transfected with 1-μg siRNA and 1-μg GFP. Recordings were performed at room temperature 24–48 h after transfection. Patch electrodes were pulled using a Flaming/Brown micropipette puller (P-1000, Sutter Instrument, CA, USA) and polished with a microforge (MF-200, WPI Inc., FL, USA). Whole-cell currents were recorded using a patch clamp amplifier (MultiClamp 700B equipped with Digidata 1440A, Molecular Devices, CA, USA). The aCSF bath solution contained (in millimole/liter) NaCl 140, CaCl_2_ 2, KCl 2, MgCl_2_ 1, glucose 20, and HEPES 20 at pH 7.4. The internal solution contained (in millimole/liter) CsCl 156, MgCl_2_ 1, EGTA 10, and HEPES 10 at pH 7.2 adjusted with CsOH [11]. NaCl was replaced with 140-mM Na-isethionate in the low-chloride aCSF. Cell recordings were performed in the presence of antagonists: 10-μM picrotoxin, 10-μM CNQX (6-cyano-7-nitoquinoxaline-2,3-dione), 50-μM D-APV (D(-)-2-amino-5-phosphonovaleric acid), 1-μM TTX (tetrodotoxin), 60-μM cadmium, and 100-μM 4AP (4-aminopyridine).

ATP depletion was induced by applying a bath solution containing 5-mM NaN_3_ and 10-mM 2-deoxyglucose (2-DG) continuously through a MicroFil (34 Gauge, WPI Inc., USA) around 10 µm away from the recording cells. The flow rate was 200 µl/min. The current–voltage relations were measured by applying voltage for 200 ms from − 80 to + 80 mV at a holding potential of − 70 mV for neurons. The sampling rate was 20 kHz and the filter setting was 1 kHz. Data were analyzed using pClamp10, version 10.2 (Molecular Devices, CA, USA).

### Reverse Transcriptase PCR

Total RNA was extracted using the TRIzol reagent (Invitrogen, Life Technologies Corporation, USA) according to the manufacturer’s protocol as previously described [10]. The primers used for SLC26A11 were as follows: 5′-CTGGAAGGTTCTGTCGCTGT-3′ (forward) and 5′-GACACCCCGGAATCACACAT-3′ (reverse).

### Lactate Dehydrogenase (LDH) Assay

Cell viability was evaluated by measuring the activity of lactate dehydrogenase (LDH) released from neurons cultured in 96-well plates, using the CytoTox 96 Non-Radioactive Cytotoxicity Assay kit (G1780, Promega, Madison, WI, USA) according to the manufacturer’s instructions. All stimulations were done after a 30-min pretreatment with 100-μM picrotoxin, 20-μM CNQX, 5-μM nifedipine (Sigma, St. Louis, MO, USA) with or without 250-μM DIDS. Neurons were stimulated by replace medium with 100-μM NMDA in aCSF or in low Cl^−^ bath solution containing above inhibitors for 30 min. Then, the solution was replaced with normal medium, and cell death was determined 24 h after the addition of NMDA; cell death is presented as the percentage of LDH released (LDH in supernatant/cell lysate LDH) × 100.

### Neurological Function Study

Motor function after MCAO was evaluated using a rotarod apparatus (Ugo Basile, Gemonio, Italy) [9, 10]. The performance of the rats was measured by observing the latency with which the rats fell off the rotarod. Before operation, the rats received 3 training trials with 15-min intervals for 5 consecutive days. The accelerating rotarod was set from 4 to 80 rpm within 10 min. The mean duration of time that the animals remained on the device was recorded 1 day before MCAO as an internal baseline control. At different time points following surgery, the mean duration of latency was recorded and compared to the internal baseline control. The Bederson scale [12] was used to measure the neurological deficits; tests include forelimb flexion, resistance to lateral push, and circling behavior with a grading scale 0–3.

### Infarct Volume Measurement

Twenty-four hours after surgery, the animals were sacrificed, and the brains were collected with cerebellum and overlying membranes being removed. The brains were sectioned into 8 slices using a brain-sectioning block, each with 2 mm in thickness. The brain slices were incubated for 30 min in a 0.1% solution of 2,3,4-triphenyltetrazolium chloride (TTC) (T4375, Sigma-Aldrich, MI, USA) at 37 °C. The sections were scanned using HP Scanjet G3110 photo scanner (HP Inc., CA, USA), and the infarct size was analyzed using ImageJ software (NIH, DC, USA). Edema-corrected lesion was calculated accordingly [9].

### Statistics

Data are expressed as the mean ± s.e.m. Statistical analyses were performed using GraphPad Prism version 6.0. Two-tailed unpaired Student’s *t* test was used to compare two means. One-way ANOVA with Bonferroni’s multiple comparison test was used to compare ≥ 3 means. Two-way ANOVA with Bonferroni’s multiple comparison test was used to analyze motor functions and time-dependent membrane capacitance change.

## Results

### Function of SLC26A11 in Neurons

The expression of SLC26A11 was first studied in healthy rat brain with immunostaining. It has been reported previously that SLC26A11 expression is high in cerebellar Purkinje neurons [13] and low in hippocampus, olfactory bulb, cerebral cortex, and subcortical structures [14]. We observed a similar staining pattern in the normal brain with a higher expression in Purkinje neurons (Supplemental Fig. 1). There is a lack of evidence supporting colocalization of SLC26A11 with GFAP in astrocytes (Supplemental Fig. 2).

**Fig. 1:**
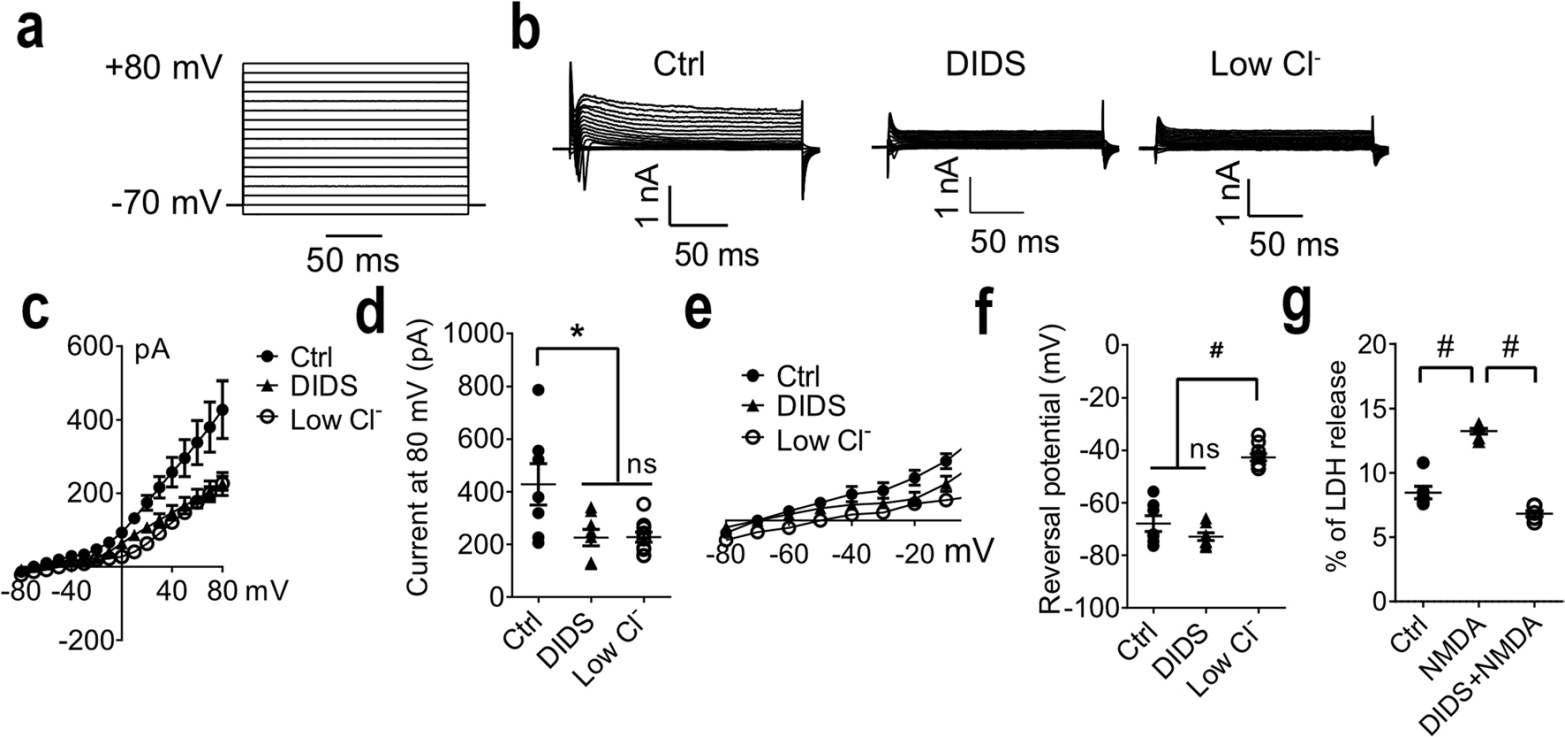
Characterization of SLC26A11 in primary cultured cortical neurons. **a** Cells were voltage clamped from − 80 to + 80 mV in steps of 10 mV at a holding potential of − 70 mV. **b** Sample currents recorded from control neurons and neurons treated with 250-μM DIDS or low chloride bath solution. **c** I-V relationship from control neurons and neurons treated with DIDS or low chloride bath solution (*n* = 7, 8, and 10). **d** Summary of currents at + 80 mV. **e** Reversal potential of control neurons and neurons receiving DIDS or low chloride bath solution. **f** Summary of reversal potential. **g** NMDAR mediated cell death measured by LDH release (*n* = 6). *P < 0.05, #*P* < 0.0001, one-way ANOVA with the Bonferroni comparison. The numerical data supporting the graphs can be found in supplemental Table S1-S3

To characterize SLC26A11 functions, whole-cell voltage clamp recordings were performed on primary cultured cortical neurons (Fig. 1a). Cl^−^ currents were recorded with GABA receptor, AMPA receptor, NMDA receptor, and other voltage-gated channels being blocked by a cocktail of blockers containing picrotoxin, CNQX (6-cyano-7-nitoquinoxaline-2,3-dione), D-APV, cadmium, and 4AP (4-aminopyridine) [8]. From a holding potential of − 70 mV, depolarization induced an outward current which can be blocked by 4,4′-diisothiocyano-2,2′-stilbenedisulfonic acid (DIDS) or a low Cl^−^ solution (Fig. 1b–d). DIDS has been reported to inhibit SLC26A11 chloride currents [8]. The low Cl^−^ solution was obtained by replacing NaCl with 140-mM Na-isethionate. When we compared the reversal potential, low Cl^−^ solution was revealed to shift the reversal potential to more positive voltage for around 30 mV compared to control neurons or DIDS-treated neurons (Fig. 1e, f). The shifting of reversal potential by low chloride solution indicates that the outward rectifying currents were carried mainly by chloride [15]. Blocking SLC26A11 with DIDS has been shown to ameliorate NMDA-induced cell death [8]. A similar effect was observed in cultured cortical neurons (Fig. 1g).

### SLC26A11 Expression Is Upregulated in Neurons After Hypoxia

**Fig. 2:**
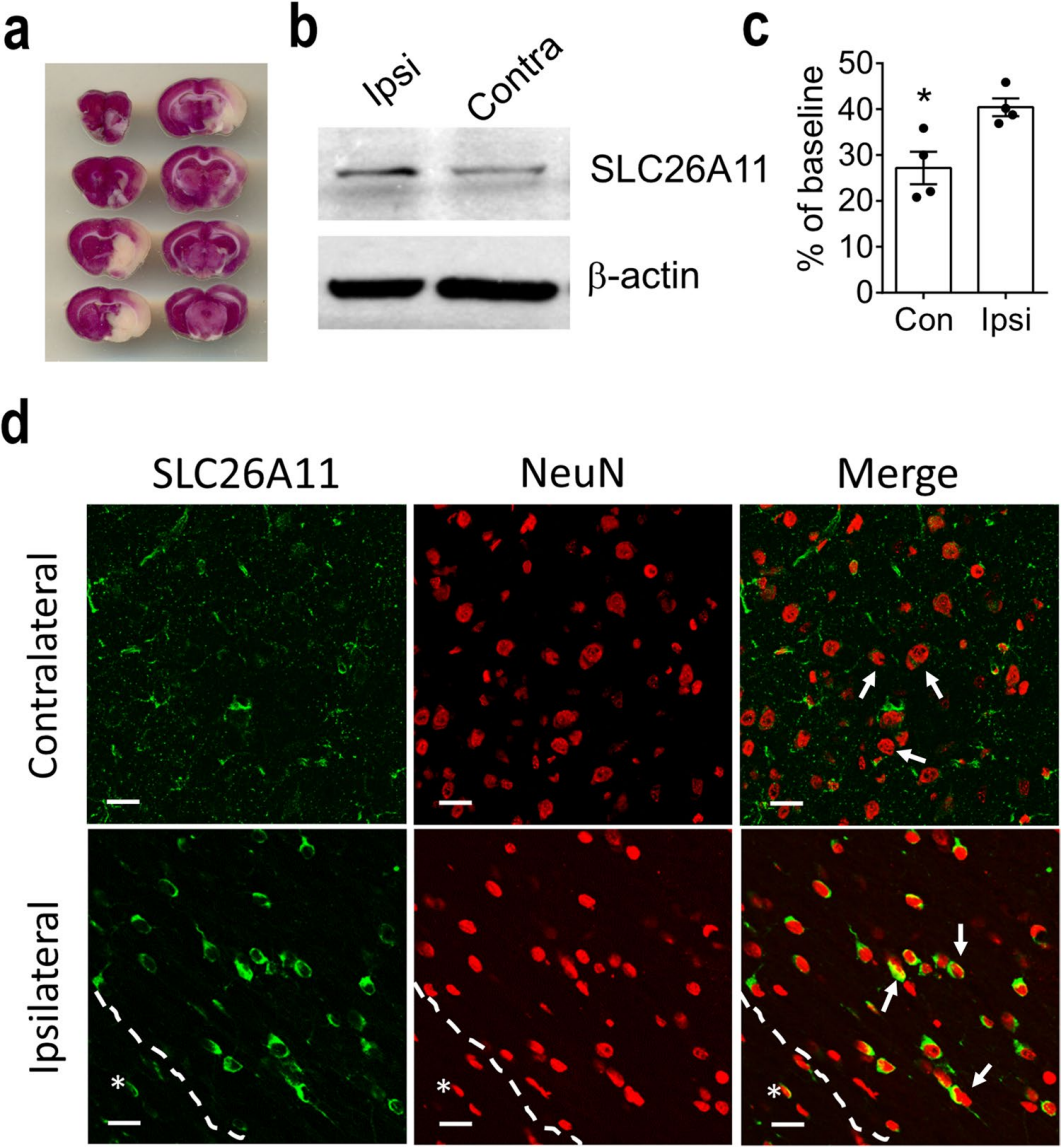
Expression of SLC26A11 in stroke rat brain. **a** TTC (2,3,5-triphenyltetrazolium chloride) staining on a rat brain with middle cerebral artery occlusion. **b** Western blot on tissues from ischemic penumbra area in ipsilateral (Ipsi) hemispheres and similar area in contralateral (Contra) hemispheres. **c** Summary of SLC26A11 expression in contralateral (Con) and ipsilateral hemispheres (Ipsi) from western blot normalized to actin. *n* = 4 experiments. *P < 0.05, Student’s *t* test. **d** Immunofluorescence staining of SLC26A11 and NeuN in contralateral and ipsilateral hemispheres. Arrows indicated neurons with representative changes of SLC26A11 expression. Asterisk: infarct core. Scale bars: 20 µm. The numerical data supporting the graphs can be found in supplemental Table S4

To examine the role of SLC26A11 under pathological conditions, we created a rat model of stroke reperfusion [9]. Middle cerebral artery was occluded for 3 h in Sprague Dawley rats, and reperfusion was achieved by removing the filament. TTC staining showed the formation of infarction (Fig. 2a). Brain tissues close to the infarct in the ipsilateral region and the corresponding region in the contralateral hemispheres were obtained for western blot (Fig. 2b). The expression of SLC26A11 was found to increase by around 48% in ipsilateral hemisphere compared to contralateral hemisphere (Fig. 2c). Immunofluorescence staining revealed that SLC26A11 was upregulated in the neurons close to the infarct, whereas the expression of SLC26A11 remained low in the contralateral hemisphere (Fig. 2d).

**Fig. 3:**
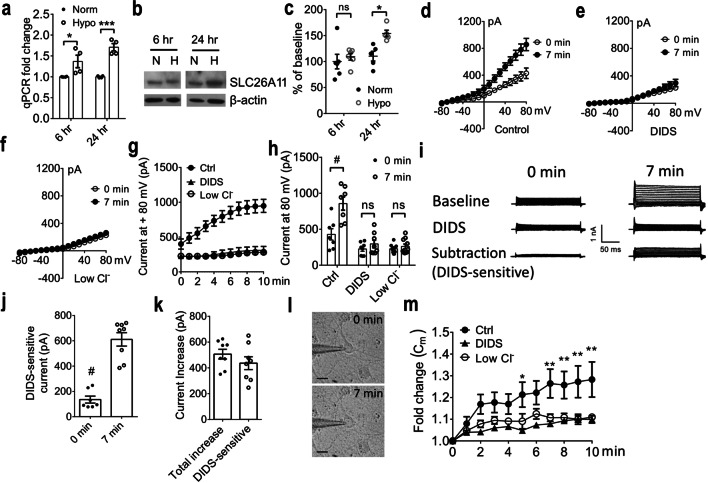
Characterization of SLC26A11 in primary cultured cortical neurons under hypoxia and ATP depletion. **a** Expression of SLC26A11 mRNA at normoxia (Norm) and at 6 h and 24 h after hypoxic treatment (Hypo) by quantitative real-time PCR. *n* = 4 experiments. **b** Western blot for SLC26A11 in control neurons under normoxia (N) and in neurons with 6-h or 24-h hypoxic treatment (H). **c** Summary of western blot normalized to SLC26A11 expression at normoxia 6 h. *n* = 6 and 5 experiments. **d** I-V relationship of control cortical neurons at baseline (0 min) and after 7-min ATP depletion. *n* = 7. **e** DIDS-treated cortical neurons before and after 7-min ATP depletion. *n* = 8. **f** I-V relationship of cortical neurons treated with low Cl^−^ bath solution at baseline (0 min) and after 7-min ATP depletion. *n* = 10. **g** Current changes in primary cultured cortical neurons after ATP depletion. For control (Ctrl) and neurons treated with DIDS or low Cl^−^ bath solution. **h** Summary of current at 80 mV. *n* = 7, 8, and 10. **i** Left: top, example traces under normal condition at 0 min. Middle, magnitude of current is reduced by DIDS. Bottom, subtraction showing DIDS-sensitive current. Right: 7-min ATP depletion increases DIDS-sensitive current. **j** Summary of DIDS-sensitive currents at + 80 mV under normal condition at 0 min and following ATP depletion at 7 min. *n* = 8. **k** Summary of overall current increase by 7-min ATP depletion at + 80 mV and DIDS-sensitive component of the current increase. *n* = 8. **l** Representative images of a neuron taken before (0 min) and after 7-min ATP depletion. **m** Comparison of membrane capacitance (C_m_) changes for control, DIDS, and low Cl.^−^-treated cortical neurons under ATP depletion induced by 5-mM NaN_3_ and 10-mM 2-DG. *n* = 10 each. In **a**, **c**, **g**, **j**, and **k**, statistical analysis was performed by Student’s* t* test and in **m** by two-way ANOVA with the Bonferroni post hoc test. *P < 0.05, **P < 0.01, ***P < 0.001, #*P* < 0.0001. The numerical data supporting the graphs can be found in supplemental Table S5-S10

SLC26A11 expression was further studied in cultured cortical neurons under hypoxic condition. Real-time PCR was first applied to examine the mRNA change upon hypoxic treatment. Compared to normoxic neurons, SLC26A11 mRNA was upregulated by 42% as early as 6 h after hypoxic induction. At 24 h, SLC26A11 mRNA was upregulated by 75% (Fig. 3a). Next, we used western blot to detect SLC26A11 protein level (Fig. 3b). At 6 h after hypoxic treatment, no difference was identified between hypoxic neurons and normoxic neurons. At 24 h after hypoxic treatment, SLC26A11 protein was increased by 40% compared to normoxic neurons (Fig. 3c).

### SLC26A11 Activity Is Enhanced Under ATP Depletion

To evaluate function of SLC26A11, electrophysiological study was performed on primary cultured cortical neurons. ATP depletion was induced by incubating the neurons with 5-mM NaN_3_ and 10-mM 2-DG. Similar as in Fig. 1, neurons under normal condition exhibited outward currents which were enhanced by 100% after 7-min ATP depletion (Fig. 3d, h). Incubation with DIDS or low chloride solution completely abolished the ATP depletion-induced current increase (Fig. 3e–h). Next, DIDS-sensitive currents were obtained by subtracting currents after DIDS treatment (Fig. 3i). Compared to normal condition at 0 min, 7-min ATP depletion significantly increased DIDS-sensitive current (Fig. 3i, j). There was no difference between the overall current increase and the DIDS-sensitive current increase induced by 7-min ATP depletion (Fig. 3i, k). This result indicates that current increase following ATP depletion is DIDS-sensitive. We have reported previously that ATP depletion could increase cell volume in neurons [4]. Here, we identified a similar increase of cell size under ATP depletion (Fig. 3l). Using cell membrane capacitance (C_m_) as a surrogate of cell volume [4], it is evidenced that ATP depletion-induced cell volume increase can be inhibited by DIDS or low chloride solution (Fig. 3m). In contrast to neurons, ATP depletion-induced current and cell volume increase were not observed in astrocytes (Supplemental Fig. 2).

Volume-regulated anion channels (VRACs) play an important role in cell volume regulation by transporting a wide variety of anions, amino acids, and organic osmolytes [16]. 4-(2-Butyl-6,7-dichloro-2-cyclopentyl-indan-1-on-5-yl) oxobutyric acid (DCPIB) is a specific VRAC inhibitor for neuroprotection in stroke [17]. In supplemental Fig. 3, application of DCPIB did not block Cl^−^ current in hypoxic neurons. DCPIB also had no effect on cell volume change during acute ATP depletion. These results suggest that VRACs are not involved in Cl^−^-associated cell volume change under current experimental conditions.

### SLC26A11-Specific siRNA Inhibits Increase of Chloride Currents Under ATP Depletion

**Fig. 4:**
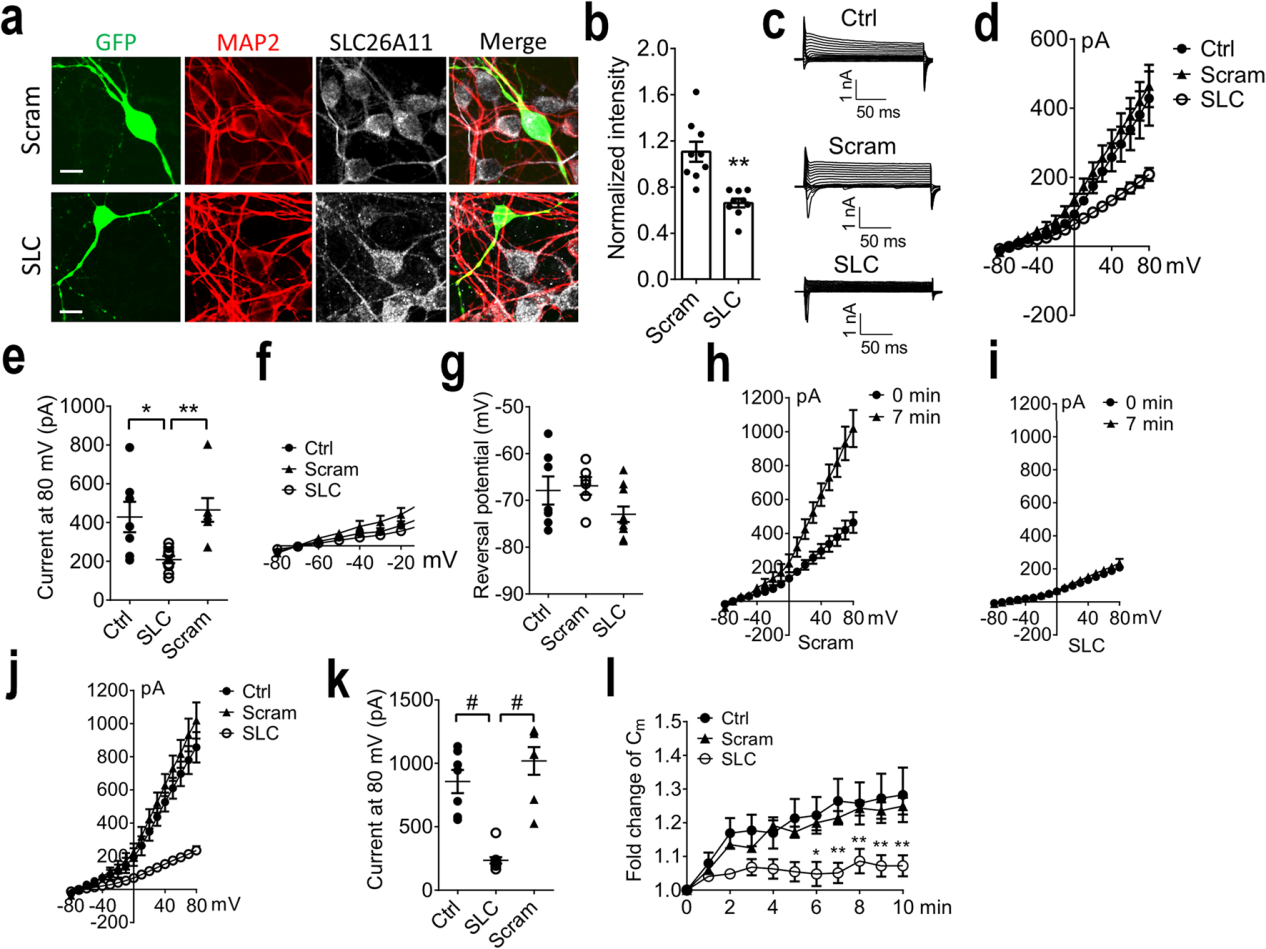
SLC26A11 inhibition by siRNA in primary cultured cortical neurons. **a** Immunofluorescence staining of neurons transfected with SLC26A11 siRNA (SLC) and control scrambled siRNA (Scram) together with GFP. Scale bars: 10 µm. **b** Comparison of SLC26A11 fluorescent intensity in GFP-positive neurons. *n* = 9 each from 3 experiments. **c** Sample currents recorded from control neurons and neurons treated with scrambled siRNA or SLC26A11-specific siRNA. Holding potential: − 70 mV. **d** I-V relationship of control neurons and neurons transfected with scrambled siRNA or SLC26A11 siRNA. *n* = 7, 7, and 10. **e** Summary of currents at + 80 mV. **f** Reversal potential of control neurons and neurons receiving scrambled siRNA or SLC26A11 siRNA. *n* = 7, 7, and 10. **g** Summary of reversal potential. **h** I-V relationship of neurons receiving scrambled siRNA at baseline (0 min) and after 7-min ATP depletion. *n* = 7. **i** I-V relationship of neurons receiving SLC26A11 siRNA at baseline (0 min) and after 7-min ATP depletion. *n* = 10. **j** Comparison of I-V relationship in control neurons and neurons receiving scrambled siRNA or SLC26A11 siRNA after 7-min ATP depletion. *n* = 7, 7, and 10. **k** Summary of currents at + 80 mV after 7-min ATP depletion. **l** Comparison of membrane capacitance (C_m_) changes for control, scrambled siRNA, and SLC26A11 siRNA-treated cortical neurons under ATP depletion induced by 5-mM NaN_3_ and 10-mM 2-DG. *n* = 10 for each group. In **b**, statistical analysis was performed by Student’s* t* test; in **e**, **g**, and **k** by one-way ANOVA with the Bonferroni post hoc test; and in **l** by two-way ANOVA with the Bonferroni post hoc test. *P < 0.05, **P < 0.01, #*P* < 0.0001. The numerical data supporting the graphs can be found in supplemental Table S11-S15

To study the SLC26A11 functions more specifically, an siRNA designed against SLC26A11 [8] was employed. GFP was co-transfected with siRNA into neurons as an indicator for successful transfection. Figure 4a shows that neurons with green fluorescence were transfected successfully. Compared to control scrambled siRNA, transfection with SLC26A11 siRNA could reduce the intensity of SLC26A11 fluorescent signal by around 40% (Fig. 4b). We further used whole-cell patch clamping method to study the effect of siRNA on GFP-positive neurons (Fig. 4c). Both scrambled siRNA and non-transfected groups exhibited similar electrophysiological properties, whereas transfection of SLC26A11 siRNA significantly inhibited channel activity (Fig. 4d). At + 80 mV, transfection of SLC26A11 siRNA could reduce the current by 50% and 34%, compared to non-transfected neurons and neurons transfected with scrambled siRNA, respectively (Fig. 4e). There is no difference between non-transfected and scrambled siRNA groups. When we examined the reversal potential (Fig. 4f), no difference was identified among the three groups (Fig. 4g).

Next, we compared the effect of siRNA on neurons with ATP depletion. In scrambled siRNA-transfected neurons, 7-min ATP depletion significantly increased channel activity (Fig. 4h) similar as in control non-transfected neurons (Fig. 3d). In sharp contrast, SLC26A11 siRNA completely suppressed current increase induced by ATP depletion (Fig. 4i). Comparing the current at + 80 mV after 7-min ATP depletion, both scrambled siRNA and control non-transfected siRNA groups exhibited similar current (Fig. 4j), whereas the current in SLC26A11 siRNA-treated neurons is significantly lower. At + 80 mV, SLC26A11 siRNA reduced the currents by 72% and 56%, compared to control non-transfected and scrambled siRNA groups, respectively (Fig. 4k). C_m_ was also recorded to examine the cell volume change. Upon ATP depletion, neurons with scrambled siRNA transfection showed similar C_m_ increase as non-transfected ones. In contrast, SLC26A11 siRNA successfully inhibited C_m_ increase induced by ATP depletion (Fig. 4l).

### SLC26A11 Inhibition Improves Stroke Outcome

**Fig. 5:**
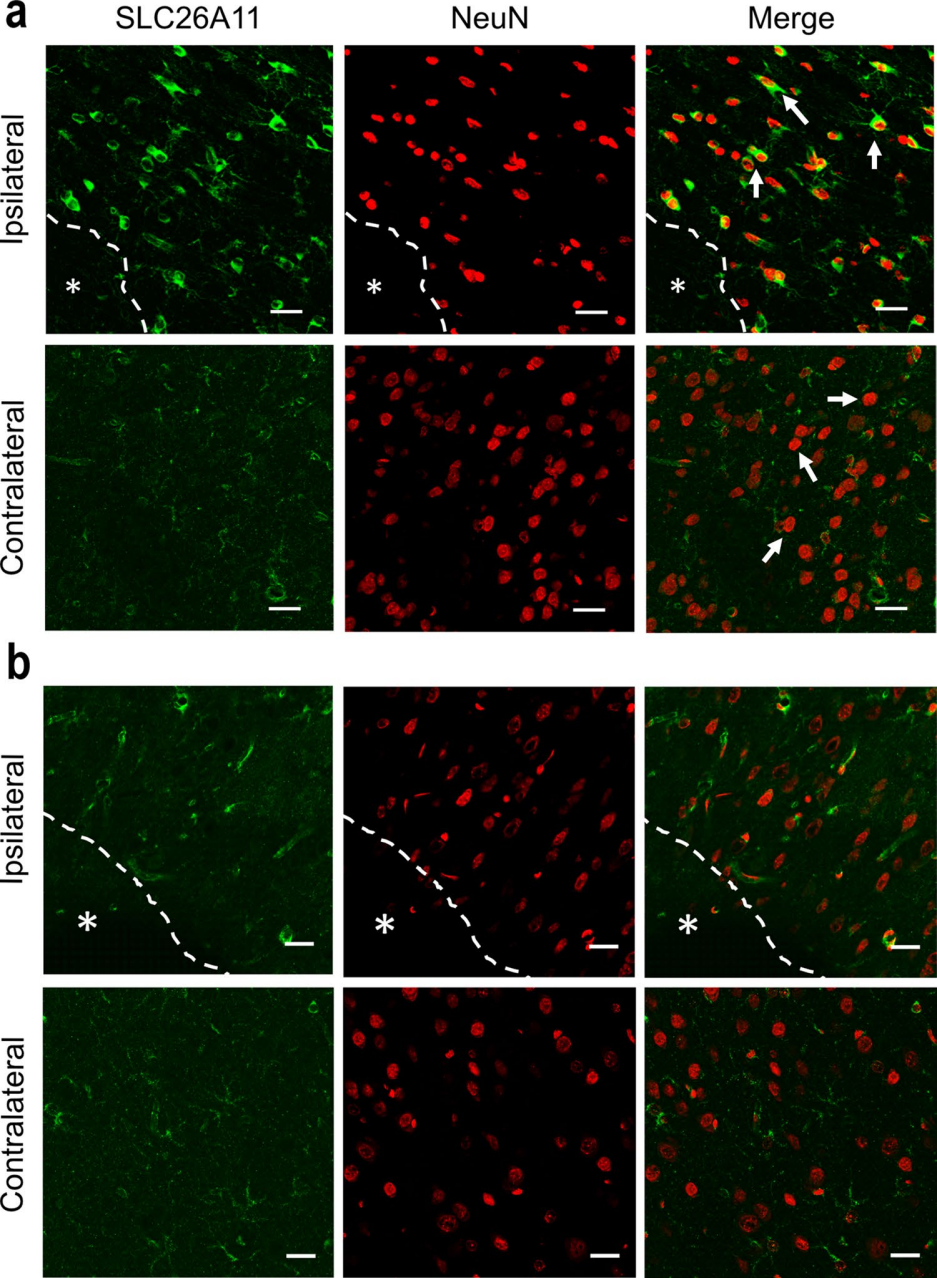
Expression of SLC26A11 in neurons was inhibited by siRNA in a rat model of stroke. **a** Immunofluorescence staining of SLC26A11 and NeuN in a rat brain receiving scrambled siRNA 1 day after stroke reperfusion. In the stroke model, middle cerebral artery was occluded for 3 h before recanalization. Asterisk: infarct core. **b** Immunofluorescence staining of SLC26A11 and NeuN in a rat brain receiving SLC26A11-specific siRNA. Asterisk: infarct core. Scale bars: 20 µm

**Fig. 6:**
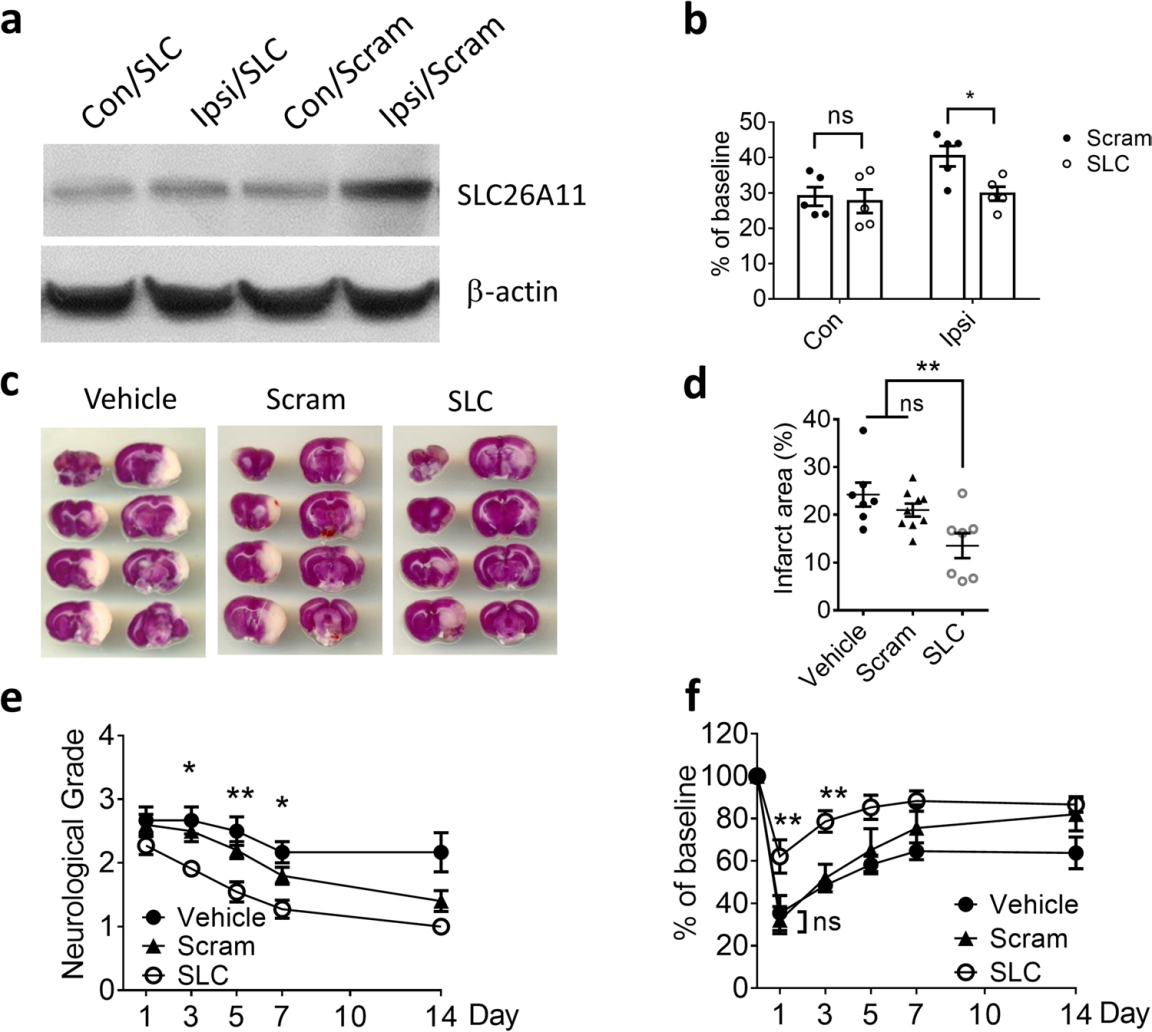
SLC26A11 inhibition on infarct volume and functional recovery after stroke. **a** Western blot on SLC26A11 expression in ischemic penumbra area in ipsilateral (Ipsi) hemispheres and similar area in contralateral (Con) hemispheres in rat brain receiving SLC26A11 siRNA (SLC) or scrambled siRNA (Scram) treatment. **b** Summary of SLC26A11 expression in rat brains after stroke. *n* = 5 rats for both groups. **c** TTC staining of rat brains receiving vehicle (Vehicle), scrambled (Scram), or SLC26A11 siRNA (SLC) treatments 1 day after stroke reperfusion. **d** Summary of infarct area. *n*_Vehicle_ = 7, *n*_Scram_ = 9 rats, and *n*_SLC_ = 7 rats. **e** Comparison of vehicle, scrambled siRNA (Scram), and SLC26A11 siRNA (SLC) treatments on the neurological severity scores in rats receiving stroke reperfusion. *n*_Vehicle_ = 6 rats, *n*_Scram_ = 10 rats, and *n*_SLC_ = 11 rats. **f** Assessment of motor functions by rotarod test. *n*_Vehicle_ = 6 rats, *n*_Scram_ = 6 rats, and *n*_SLC_ = 7 rats. In **b**, statistical analysis was performed by Student’s* t* test and in **d**, **e**, and **f** by two-way ANOVA with Bonferroni post hoc test. *P < 0.05, **P < 0.01, SLC vs Vehicle or Scram. No significant difference was detected between Vehicle and Scram at indicating data point. The numerical data supporting the graphs can be found in supplemental Table S16-S19

To examine the in vivo effect of SLC26A11, a stroke reperfusion model was established in rats in which middle cerebral arteries were transiently occluded for 3 h [18]. The 25-nmol siRNA was injected into rats after occlusion. We have used the same dose of siRNA to inhibit TRPM4 channel in a similar stroke reperfusion model [9]. Immunofluorescence staining was performed to examine the expression of SLC26A11. The staining pattern in scrambled siRNA-treated brains (Fig. 5a) is similar to Fig. 2, in which SLC26A11 is prominently expressed in neurons close to the infarct core within the ipsilateral hemisphere, whereas weak staining was observed in the contralateral hemisphere (Fig. 5a). In animals receiving SLC26A11-specific siRNA, neurons close to the infarct core were negatively stained for SLC26A11 (Fig. 5b), suggesting that siRNA has successfully inhibited SLC26A11 upregulation in neurons after stroke. Western blot further verified the staining result (Fig. 6a). In scrambled siRNA-treated animals, the expression of SLC26A11 in ipsilateral hemisphere is 34.6% higher than contralateral hemisphere (Fig. 6b). Application of SLC26A11 siRNA completely inhibited the upregulation in ipsilateral hemisphere. There is no difference between the ipsilateral and contralateral hemispheres (Fig. 6b).

When the infarct volume was examined 24 h after operation, we found that the infarct volume is clearly smaller in animals receiving SLC26A11 siRNA (Fig. 6c). Compared to scrambled siRNA and vehicle application, SLC26A11 siRNA could reduce the infarct volume significantly by more than 35% (Fig. 6d). In scrambled siRNA-treated brains, infarction occupies around 21% of total brain volume, whereas SLC26A11 siRNA-treated brains have around 13.6% of total brain volume being infarcted. Neurological deficit following stroke was examined using a neurological severity score system [12]. Both scrambled siRNA and SLC26A11 siRNA treatments, as well as vehicle application, have demonstrated improvement in neurological functions following stroke (Fig. 6e). Compared to vehicle and scrambled siRNA, animals receiving SLC26A11 siRNA have shown a faster functional recovery. Motor functions were further evaluated using a rotarod apparatus [19]. At days 1 and 3 after stroke, animals receiving vehicle and scrambled siRNA exhibited a more significant drop of motor functions compared to animals receiving SLC26A11 siRNA treatment (Fig. 6f).

## Discussion

Our results demonstrate that SLC26A11 plays an important role in hypoxia-induced neuronal oncosis. Blocking SLC26A11 could attenuate cerebral injury after stroke reperfusion. SLC26A11 has been shown to conduct Cl^−^ influx following prolonged Na^+^ entry [8]. In this study, SLC26A11 activity was revealed to be increased in neurons following hypoxia. First, hypoxia enhances SLC26A11 expression in neurons. SLC26A11 mRNA was upregulated as early as 6 h after hypoxic induction (Fig. 3), and later, the protein level was elevated accordingly. In a rat stroke reperfusion model, SLC26A11 was mainly located in surviving neurons close to the infarct core, suggesting that the hypoxic environment could induce SLC26A11 expression. Second, membrane depolarization in neurons could enhance SLC26A11 activity. It is likely that the membrane depolarization during ATP depletion creates a greater driving force for Cl^−^ entry. This is supported by the previous study indicating that membrane depolarization to − 20 mV or greater activates SLC26A11 [8]. Furthermore, being first discovered as a sulfate transporter [20], membrane depolarization could activate SLC26A11 in its Cl^−^ channel mode [8, 14]. Our results on primary cultured cortical neurons support this finding. At a more positive membrane potential, the outward rectifying current in cortical neurons becomes more prominent which is blocked by DIDS, low Cl solution, or SLC26A11 siRNA (Figs. 1 and 4). Thus, ATP depletion could greatly enhance this outward rectifying current (Figs. 3 and 4). As hypoxia-induced Cl^−^ entry was completely abolished by SLC26A11 siRNA in cultured cortical neurons under our experimental conditions, Na^+^-K^+^-2Cl^−^ co-transporter 1 (NKCC1) [1] does not contribute to Cl^−^ entry without β-adrenergic stimulation. We also did not observe a contribution of Cl^−^ entry from volume-regulated anion channels (VRACs) in cultured cortical neurons (supplemental Fig. 3). However, in animal stroke model, these Cl^−^ entry pathways can be activated with relevant activators.

Rungta et al. have found that SLC26A11-conducted Cl^−^ influx is associated with Na^+^ entry via prolonged activation of VSSC or NMDA receptors [8]. In this study, ATP depletion in neurons was induced by metabolic inhibitors 2-DG and NaN_3_ which disrupt glycolysis and oxidative phosphorylation [21, 22]. No activators for VSCC or NMDA receptors were applied. Acid-sensing ion channels (ASICs) [3] were also inactivated as the solution does not contain a high level of proton. Therefore, there exists additional source of Na^+^ ions that is associated with SLC26A11-conducted Cl^−^ influx. It has been reported previously that TRPM4 channel is a major source of Na^+^ entry under the same condition [23]. Similar as SLC26A11, TRPM4 expression is low in healthy brain and upregulated after hypoxic induction [24]. Interestingly, the expression pattern of TRPM4 in neurons after stroke is also similar as SLC26A11 [9, 10], mainly located within the surviving tissue close to the infarct core. Furthermore, blocking TRPM4 is known to inhibit hypoxia-induced oncosis [4, 23], an effect close to the blockade of SLC26A11 (Figs. 3 and 4). The mechanism for SLC26A11 and TRPM4 upregulation remains unknown. It has been reported that mTOR pathway is activated in ischemic penumbra and suppressed in ischemic core [25]. Whether mTOR activity is associated with the expression of SLC26A11 and TRPM4 needs further investigation.

**Fig. 7:**
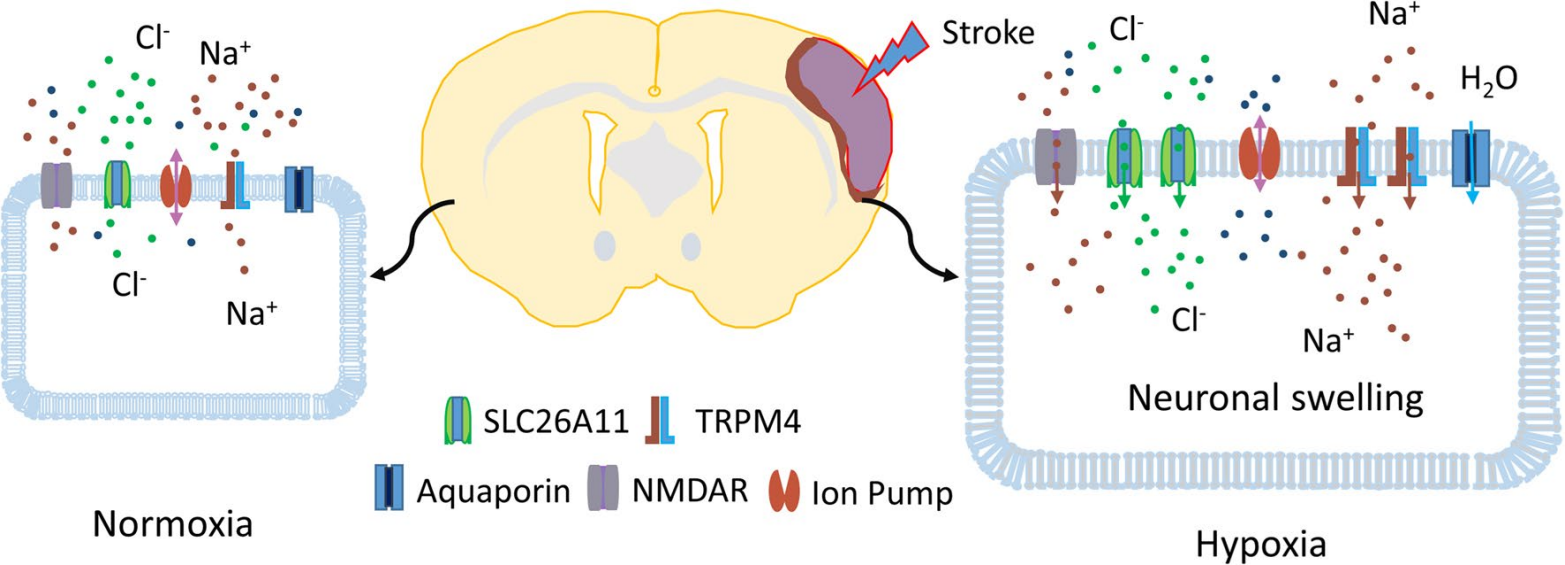
Mechanism of neuronal oncosis in stroke. In healthy neurons, transmembrane movement of Na^+^ and Cl^−^ ions is are highly controlled, maintaining the normal cell volume and shape. After stroke, hypoxia induces many changes to the neurons including ATP depletion, increase of intracellular Ca^2+^ concentration, and membrane depolarization. Na^+^ permeable channels such as TRPM4 channel and NMDA receptors are activated, conducting excessive Na^+^ entry. With the driving force of Cl^−^ ions increases, Cl^−^ influx via SLC26A11 is enhanced to balance the intracellular positively charged Na^+^ ions, leading to a higher concentration of intracellular NaCl. As a result, osmotic pressure is elevated, causing water influx via aquaporins and subsequent cell volume increase

TRPM4 is a monovalent cation channel. Under hypoxia, TRPM4 can be activated by ATP depletion and intracellular Ca^2+^ accumulation [26]. Na^+^ entry via TRPM4 also depolarizes membrane potential. Cl^−^ current fluxes via SLC26A11 thus balance the elevated intracellular Na^+^ ions to reach electroneutrality, helping repolarize membrane potential. Additionally, accumulation of intracellular acid by hypoxia could possibly facilitate SLC26A11 conducting Cl^−^ entry. In the kidney, chloride flux via SLC26A11 has been shown to increase acid extrusion by H^+^-ATPase in the collecting tube [27]. It was suggested that the chloride secretion via SLC26A11 helps to dissipate the lumen-positive voltage causing by H^+^ secretion. At cellular level, proteins contribute to the negative charge on the intracellular side of the membrane. However, due to the large size, transmembrane movement is difficult and less efficient under hypoxia [28]. Therefore, Cl^−^ becomes the major negatively charged ions moving across cell membrane. Net influx of Na^+^ and Cl^−^ during hypoxia inevitably increases intracellular osmotic pressure, thereby leading to oncosis. Thus, blocking Na^+^ and Cl^−^ during hypoxia can protect neurons after hypoxic diseases such as stroke. In this study, Na^+^ entry pathway such as TRPM4 was not blocked. However, blocking Cl^−^ influx alone could ameliorate cellular swelling, suggesting that oncosis requires the entry of both Na^+^ and Cl^−^ ions together. Being a sulfate transporter [20], transient inhibition of SLC26A11 is unlikely to severely disrupt sulfate uptake in this study. Other sulfate transporters such as SLC26A2 may play a more important role in sulfate metabolism. For example, inactivation of SLC26A2 depletes intracellular sulfate and leads to the synthesis of undersulfated proteoglycans in susceptible cells such as chondrocytes and fibroblasts [29]. The possible mechanisms of neuronal oncosis in stroke are summarized in Fig. 7.

In this study, in vivo application of SLC26A11 siRNA resulted in a reduction of protein expression within the ipsilateral hemisphere. SLC26A11 expression in contralateral hemisphere remained unchanged (Figs. 5 and 6). This in vivo siRNA effect targeting hypoxic tissue was reported in other studies on stroke. We have found that TRPM4 siRNA could suppress TRPM4 expression only within the ipsilateral hemisphere in both permanent and transient stroke models [9, 10]. The most possible reason is that stroke disrupts the blood–brain barrier (BBB) within the ipsilateral hemisphere which facilitates the entry of siRNA into the brain parenchyma. On the other hand, the expression of SLC26A11 and TRPM4 is low in the healthy neurons where the protein turnover rates are low. One exception is the higher expression of SLC26A11 in cerebellar Purkinje cells [13]. The SLC26A11 siRNA unlikely affects cerebellar functions as the brain area is protected by BBB, and also, the effect of single-dose siRNA is transient. In ipsilateral hemisphere, hypoxia greatly increases the transcription of SLC26A11 and TRPM4. Therefore, siRNA could enter the brain via disrupted BBB to achieve a more prominent suppressive outcome. It should be noted that siRNA needs to be administered before transcript upregulation to suppress protein expression. Therefore, it remains a challenge to apply siRNA at very early stage in stroke patients. A direct blocker to SLC26A11 will achieve a better therapeutic potential.

Neuronal death occurs immediately after stroke onset, participating in the formation of infarct core [30]. Tissues surrounding the infarct core are known as penumbra. Neurons within this area have a chance to survive if reperfusion is achieved in time. Identification of the role of SLC26A11 in neurons within the penumbra area is critical for neuroprotection. Reduction of oncosis by blocking SLC26A11 or Na^+^ entry pathways could extend the survival time for neurons. More neurons can thus be rescued when reperfusion is achieved. Unlike NMDA receptors which are ubiquitously expressed in the brain, the expression of SLC26A11 and TRPM4 is low in healthy neurons in most brain regions. Their pathophysiological roles in stroke are mainly from their upregulated expression and enhanced activity created by the hypoxic environment. Therefore, blocking SLC26A11 or TRPM4 is unlikely to generate side effects on healthy neurons that have been found in NMDA blocker application [31]. It would be interesting to investigate whether blocking Na^+^ and Cl^−^ entry together would generate a synergistic effect on inhibiting reperfusion injury [32] or improving learning and memory [33] after stroke.

In conclusion, we have identified that SLC26A11 is important for neuronal oncosis following hypoxia and stroke. Blocking SLC26A11 could achieve neuroprotection and improve functional outcome in stroke reperfusion.

## Supplementary Information

Below is the link to the electronic supplementary material.

Supplementary file1 (DOCX 46 KB)

Supplemental Fig. 1 (PNG 13149 kb)

High resolution image (TIF 3397 kb)

Supplemental Fig. 2 (PNG 639 kb)

High resolution image (TIF 1185 kb)

Supplemental Fig. 3 (PNG 110 kb)

High resolution image (TIF 490 kb)

## Data Availability

All data generated and analyzed for this study are included in this published article and its supplementary additional files.

## References

[1] Loh KY, Wang Z, Liao P (2019). Oncotic cell death in stroke. Rev Physiol Biochem Pharmacol.

[2] Choi DW (1987). Ionic dependence of glutamate neurotoxicity. J Neurosci.

[3] Xiong ZG (2008). Acid-sensing ion channels (ASICs) as pharmacological targets for neurodegenerative diseases. Curr Opin Pharmacol.

[4] Wei S (2020). Comparison of anti-oncotic effect of TRPM4 blocking antibody in neuron, astrocyte and vascular endothelial cell under hypoxia. Front Cell Dev Biol.

[5] Pivonkova H (2018). The contribution of TRPV4 Channels to astrocyte volume regulation and brain edema formation. Neuroscience.

[6] Banasiak KJ, Burenkova O, Haddad GG (2004). Activation of voltage-sensitive sodium channels during oxygen deprivation leads to apoptotic neuronal death. Neuroscience.

[7] Kahle KT (2009). Molecular mechanisms of ischemic cerebral edema: role of electroneutral ion transport. Physiology (Bethesda).

[8] Rungta RL (2015). The cellular mechanisms of neuronal swelling underlying cytotoxic edema. Cell.

[9] Chen B (2019). Non-invasive multimodality imaging directly shows TRPM4 inhibition ameliorates stroke reperfusion injury. Transl Stroke Res.

[10] Loh KP (2014). TRPM4 inhibition promotes angiogenesis after ischemic stroke. Pflugers Arch.

[11] Schattling B (2012). TRPM4 cation channel mediates axonal and neuronal degeneration in experimental autoimmune encephalomyelitis and multiple sclerosis. Nat Med.

[12] Bederson JB (1986). Rat middle cerebral artery occlusion: evaluation of the model and development of a neurologic examination. Stroke.

[13] Rahmati N et al (2016) SLC26A11 (KBAT) in Purkinje cells is critical for inhibitory transmission and contributes to locomotor coordination. eNeuro 3(3):ENEURO.0028-16.2016 10.1523/ENEURO.0028-16.2016 PMC4908300 27390771

[14] Rahmati N (2013). Slc26a11 is prominently expressed in the brain and functions as a chloride channel: expression in Purkinje cells and stimulation of V H(+)-ATPase. Pflugers Arch.

[15] Wang M (2012). Downregulation of TMEM16A calcium-activated chloride channel contributes to cerebrovascular remodeling during hypertension by promoting basilar smooth muscle cell proliferation. Circulation.

[16] Nilius B (2004). Is the volume-regulated anion channel VRAC a “water-permeable” channel?. Neurochem Res.

[17] Zhang Y (2008). DCPIB, a specific inhibitor of volume regulated anion channels (VRACs), reduces infarct size in MCAo and the release of glutamate in the ischemic cortical penumbra. Exp Neurol.

[18] Chen B (2019). TRPM4-specific blocking antibody attenuates reperfusion injury in a rat model of stroke. Pflugers Arch.

[19] Low SW (2021). Development and characterization of a monoclonal antibody blocking human TRPM4 channel. Sci Rep.

[20] Vincourt JB (2003). Molecular and functional characterization of SLC26A11, a sodium-independent sulfate transporter from high endothelial venules. FASEB J.

[21] Tsubaki M (1993). Fourier-transform infrared study of cyanide binding to the Fea3-CuB binuclear site of bovine heart cytochrome c oxidase: implication of the redox-linked conformational change at the binuclear site. Biochemistry.

[22] Horton RW, Meldrum BS, Bachelard HS (1973). Enzymic and cerebral metabolic effects of 2-deoxy-D-glucose. J Neurochem.

[23] Gerzanich V (2009). De novo expression of Trpm4 initiates secondary hemorrhage in spinal cord injury. Nat Med.

[24] Ahad MA (2020). Insights into the neuropathology of cerebral ischemia and its mechanisms. Rev Neurosci.

[25] Wu M (2018). Rapamycin prevents cerebral stroke by modulating apoptosis and autophagy in penumbra in rats. Ann Clin Transl Neurol.

[26] Mathar I (2014). Trpm4. Handb Exp Pharmacol.

[27] Xu J (2011). Slc26a11, a chloride transporter, localizes with the vacuolar H(+)-ATPase of A-intercalated cells of the kidney. Kidney Int.

[28] Hansen AJ (1985). Effect of anoxia on ion distribution in the brain. Physiol Rev.

[29] Rossi A, Superti-Furga A (2001). Mutations in the diastrophic dysplasia sulfate transporter (DTDST) gene (SLC26A2): 22 novel mutations, mutation review, associated skeletal phenotypes, and diagnostic relevance. Hum Mutat.

[30] Lipton P (1999). Ischemic cell death in brain neurons. Physiol Rev.

[31] Wu QJ, Tymianski M (2018). Targeting NMDA receptors in stroke: new hope in neuroprotection. Mol Brain.

[32] Chen B (2023). TRPM4 blocking antibody protects cerebral vasculature in delayed stroke reperfusion. Biomedicines.

[33] Hazalin N, Liao P, Hassan Z (2020). TRPM4 inhibition improves spatial memory impairment and hippocampal long-term potentiation deficit in chronic cerebral hypoperfused rats. Behav Brain Res.

