# Supplementary material for: SLC26A11 Inhibition Reduces Oncotic Neuronal Death and Attenuates Stroke Reperfusion Injury
Source: Mol Neurobiol. 2023 Jun 28;60(10):5931–43. doi: 10.1007/s12035-023-03453-1 (PMC10471688; doi:10.1007/s12035-023-03453-1)
Supplement: Supplementary file 1 — Supplementary file1 (DOCX 46 KB) [file 12035_2023_3453_MOESM1_ESM.docx]

**Supplementary Information**

**SLC26A11 inhibition reduces oncotic neuronal death and attenuates stroke reperfusion injury**

Shunhui Wei^1†^, PhD; Bo Chen^1†^, PhD; See Wee Low^1^, MS; Charlene Priscilla Poore^1^, PhD; Yahui Gao^1‡^, MS; Bernd Nilius^2^, MD, PhD; Ping Liao^1,3,4^, MD, PhD

^1^Calcium Signalling Laboratory, Department of Research, National Neuroscience Institute, Singapore 308433, Singapore,

^2^Department of Cellular and Molecular Medicine, KU Leuven, Leuven 3000, Belgium,

^3^Duke-NUS Medical School, Singapore 169857, Singapore,

^4^Health and Social Sciences, Singapore Institute of Technology, Singapore 138683, Singapore.

^†^These authors contributed equally to this work.

^‡^Current affiliation: Department of Chemical and Biomolecular Engineering, National university of Singapore, Singapore 119077, Singapore

Correspondence to: Ping Liao, MD, PhD. Calcium Signalling Lab, National Neuroscience Institute, 11 Jalan Tan Tock Seng, Singapore 308433. Fax: (65) 6256 9178; Tel: (65) 6357 7611; E-mail: [gmslip@nus.edu.sg](mailto:gmslip@nus.edu.sg)

**Materials and methods**

**Immunofluorescence staining**

Rats were sacrificed and perfused and the brains were harvested and sectioned at 10 µm in thickness. Following fixation with 4% paraformaldehyde, the brain slice was incubated in 100 μl blocking serum (10 % fetal bovine serum in 0.2 % PBST) for 1 hr. Primary antibodies include Anti-SLC26A11 (HPA029893, Sigma-Aldrich, MO, USA. 1:100), anti-NeuN (MAB377, Millipore, MA, USA, 1:250), anti-GFAP (IF03L, Millipore, MA, USA, 1:200), Secondary antibodies are conjugated with FITC or Alexa Fluor 594.

**Primary rat astrocytes culture**

For primary culture of cortical astrocytes, cells from cerebral cortex were digested, dissociated, and maintained for 10 days in DMEM supplemented with 10% FBS. Cultures were then treated with 10 µM Ara-C, shaken at 240 rpm for 6 hrs to remove oligodendrocyte precursor cells and replanted for experiments.

**Fig. S1** Expression of SLC26A11 in neurons of rat brain. Immunofluorescence staining of SLC26A11 and NeuN in rat brain hippocampus (upper panel), cortex (middle panel), cerebellum (lower panel). Scale bars: 20 µm.

**Fig. S2** Characterization of SLC26A11 in primary cultured astrocytes under ATP-depletion. **a** Expression of SLC26A11 in astrocytes in cortex of rat brain. Immunofluorescence staining of SLC26A11 and GFAP. Scale bars: 20 µm. **b** I-V relationship of control astrocytes (Ctrl) at baseline (0 min) and after 7-min ATP-depletion. n = 7. **c** I-V relationship of astrocytes transfected with SLC26A11 siRNA (SLC). n = 8. **d** I-V relationship of astrocytes transfected with scrambled siRNA (Scram), n = 8. **e** DIDS treated astrocytes before and after 7-min ATP-depletion. n =7. **f** Summary of current at 80 mV at baseline (0 min) and at 7 min after ATP-depletion. n = 7, 8, 8 and 7. **g** Comparison of membrane capacitance (Cm) changes for control, SLC26A11 siRNA (SLC), Scramble siRNA (Scram) and DIDS treated astrocytes after ATP-depletion induced by 5 mM NaN3 and 10 mM 2-DG. n = 7, 8, 8 and 7.

**Fig. S3** DCPIB does not affect ATP-depletion increased current in neurons. **a** I-V relationship of neurons receiving DCPIB (10 μM) treatment at baseline (0 min) and after 7-min ATP-depletion. *n* = 7. **b** Comparison of I-V relationship in control neurons (Ctrl) and neurons receiving DCPIB treatment (DCPIB) after 7-min ATP-depletion. *n* = 7, 7. **c** Comparison of membrane capacitance (C_m_) changes for control (Ctrl) and DCPIB treated neurons under ATP-depletion induced by 5 mM NaN_3_ and 10 mM 2-DG. *n* = 10, 7 for each group.

**Table S1** Summary of currents at +80 mV (Fig. 1**d**)

| Summary of current at + 80 mV | | | |
| --- | --- | --- | --- |
| N | **Treatment** | **Current (pA)** | ***p*-Value** |
| 7 | Ctrl | 428.0 ± 78.41 | 0.0133, F (2, 22) = 6.491  Vs. DIDS, Low Cl^-^ |
| 8 | DIDS | 225.5 ± 31.32 |  |
| 10 | Low Cl^-^ | 227.5 ± 19.18 |  |

Data are presented as mean ± S.E.M

**Table S2** Summary of reversal potential (Fig. 1**f**)

| Summary of reversal potential | | | |
| --- | --- | --- | --- |
| N | **Treatment** | **Reversal potential (mV)** | ***p*-Value** |
| 7 | Ctrl | -67.90 ± 3.01 |  |
| 8 | DIDS | -72.89 ± 1.45 |  |
| 10 | Low Cl^-^ | -42.67 ± 1.39 | < 0.0001, F (2, 22) = 77.85  vs. DIDS, Ctrl |

Data are presented as mean ± S.E.M

**Table S3** NMDAR mediated cell death measured by LDH release (Fig. 1**g**)

| Summary of LDH release | | | |
| --- | --- | --- | --- |
| N | **Treatment** | **LDH release (%)** | ***p*-Value** |
| 6 | Ctrl | 9.19 ± 0.40 |  |
| 6 | NMDA | 13.25 ± 0.22 | < 0.0001, F (2, 15) = 122.7  vs. Ctrl, DIDS + NMDA |
| 6 | NMDA + DIDS | 6.82 ± 0.22 |  |

Data are presented as mean ± S.E.M

**Table S4** Summary of SLC26A11 expression (Fig. 2**c**)

| Summary of SLC26A11 expression | | | |
| --- | --- | --- | --- |
| N | **Treatment** | **Percentage of**  **b-actin (%)** | ***p*-Value** |
| 4 | Con | 27.19 ± 3.53 | 0.0212 vs.Ipsi |
| 4 | Ipsi | 40.42 ± 1.93 |  |

Data are presented as mean ± S.E.M

**Table S5** Expression of SLC26A11 mRNA (Fig. 3**a**)

| Expression of SLC26A11 mRNA | | | | | | |
| --- | --- | --- | --- | --- | --- | --- |
| Normoxia | | | | | **Hypoxia** | |
| N | **Fold change** | **Treatment** | **N** | **Fold change** | | ***p*-Value vs Normoxia** |
| 4 | 1.00 ± 0.04 | 6 hr | 4 | 1.42 ± 0.14 | | <0.05 |
| 4 | 1.00 ± 0.12 | 24 hr | 4 | 1.76 ± 0.08 | | <0.001 |

Data are presented as mean ± S.E.M

**Table S6** Summary of SLC26A11 expression level (Fig. 3**c**)

| Summary of SLC26A11 expression level | | | | | | |
| --- | --- | --- | --- | --- | --- | --- |
| Normoxia | | | | | **Hypoxia** | |
| N | **% of baseline** | **Treatment** | **N** | **% of baseline** | | ***p*-Value vs Normoxia** |
| 6 | 92.26 ± 16.32 | 6 hr | 6 | 109.30 ± 6.43 | | 0.062 |
| 5 | 100.50 ± 9.36 | 24 hr | 5 | 154.10 ± 6.40 | | 0.0049 |

Data are presented as mean ± S.E.M

**Table S7** Current changes in neurons after ATP-depletion (Fig. 3**h**)

| Summary of current at 80 mV | | | | | | |
| --- | --- | --- | --- | --- | --- | --- |
| Normoxia | | | | | **ATP depletion 7 min** | |
| N | **Current (pA)** | **Treatment** | **N** | **Current (pA)** | | ***p*-Value vs Normoxia** |
| 7 | 427.98 ± 78.41 | Ctrl | 7 | 856.65 ± 91.57 | | < 0.0001 |
| 8 | 225.52 ± 31.32 | DIDS | 8 | 295.55 ± 53.11 | | 0.275 |
| 10 | 227.54 ± 19.18 | Low Cl^-^ | 10 | 261.24 ± 32.34 | | 0.382 |

Data are presented as mean ± S.E.M

**Table S8** Summary of DIDS-sensitive currents (Fig. 3**j**)

| Summary of DIDS-sensitive currents at + 80 mV | | | |
| --- | --- | --- | --- |
| N | **ATP depletion (min)** | **Current (pA)** | ***p*-Value** |
| 8 | 0 | 174.60 ± 45.45 | < 0.0001 vs. 7 min |
| 8 | 7 | 611.10 ± 52.65 |  |

Data are presented as mean ± S.E.M

**Table S9** Summary of current increase by 7-min ATP-depletion (Fig. 3**k**)

| Summary of current increase at + 80 mV | | | |
| --- | --- | --- | --- |
| N | **Condition** | **Current (pA)** | ***p*-Value** |
| 8 | Total | 506.60 ± 37.59 | 0.2801 |
| 8 | DIDS-sensitive | 436.6 ± 49.71 |  |

Data are presented as mean ± S.E.M

**Table S10** Comparison of membrane capacitance (Cm) changes (Fig. 3**m**)

| Comparison of membrane capacitance (Cm) changes | | | | |
| --- | --- | --- | --- | --- |
| N | **Treatment** | **ATP depletion (min)** | **Fold change** | ***p*-Value** |
| 10 | Ctrl | 5 | 1.21 ± 0.06 | <0.038 vs. DIDS, Low Cl^-^ |
| 10 | DIDS | 5 | 1.05 ± 0.01 |  |
| 10 | Low Cl^-^ | 5 | 1.09 ± 0.03 |  |
| 10 | Ctrl | 7 | 1.26 ± 0.07 | <0.0042 vs. DIDS, Low Cl^-^ |
| 10 | DIDS | 7 | 1.07 ± 0.01 |  |
| 10 | Low Cl^-^ | 7 | 1.11 ± 0.02 |  |
| 10 | Ctrl | 8 | 1.26 ± 0.06 | <0.0073 vs. DIDS, Low Cl^-^ |
| 10 | DIDS | 8 | 1.08 ± 0.02 |  |
| 10 | Low Cl^-^ | 8 | 1.11 ± 0.02 |  |
| 10 | Ctrl | 9 | 1.27 ± 0.07 | <0.0015 vs. DIDS, Low Cl^-^ |
| 10 | DIDS | 9 | 1.09 ± 0.02 |  |
| 10 | Low Cl^-^ | 9 | 1.10 ± 0.02 |  |
| 10 | Ctrl | 10 | 1.28 ± 0.08 | <0.0014 vs. DIDS, Low Cl^-^ |
| 10 | DIDS | 10 | 1.09 ± 0.02 |  |
| 10 | Low Cl^-^ | 10 | 1.11 ± 0.02 |  |

Data are presented as mean ± S.E.M

**Table S11** Comparison of SLC26A11 fluorescent intensity (Fig. 4**b**)

| Comparison of SLC26A11 fluorescent intensity | | | |
| --- | --- | --- | --- |
| N | **Treatment** | **Normalized intensity** | ***p*-Value** |
| 9 | Scram | 1.00 ± 0.078 | 0.0002 |
| 9 | SLC | 0.60 ± 0.035 |  |

Data are presented as mean ± S.E.M

**Table S12** Summary of currents at +80 mV (Fig. 4**e**)

| Summary of current at + 80 mV | | | |
| --- | --- | --- | --- |
| N | **Treatment** | **Current (pA)** | ***p*-Value** |
| 7 | Ctrl | 428.0 ± 78.41 |  |
| 10 | SLC | 208.3 ± 18.54 | <0.0028 vs. Ctrl, Scram |
| 7 | Scram | 464.3 ± 61.23 |  |

Data are presented as mean ± S.E.M

**Table S13** Summary of reversal potential (Fig. 4**g**)

| Summary of reversal potential | | | |
| --- | --- | --- | --- |
| N | **Treatment** | **Reversal potential (mV)** | ***p*-Value** |
| 7 | Ctrl | -67.90 ± 3.012 |  |
| 10 | SLC | -72.98 ± 1.660 | 0.0666, F (2, 21) = 3.091 |
| 7 | Scram | -62.51 ± 4.669 |  |

Data are presented as mean ± S.E.M

**Table S14** Summary of currents at + 80 mV after 7-min ATP-depletion (Fig. 4**k**)

| Summary of current at + 80 mV | | | |
| --- | --- | --- | --- |
| N | **Treatment** | **Current (pA)** | ***p*-Value** |
| 7 | Ctrl | 856.60 ± 91.57 |  |
| 10 | SLC | 235.00 ± 24.91 | P < 0.0001, F (2, 21) = 34.35 vs. Ctrl, Scram |
| 7 | Scram | 1019.00 ± 109.20 |  |

Data are presented as mean ± S.E.M

**Table S15** Comparison of membrane capacitance (Cm) changes (Fig. 4**l**)

| Comparison of membrane capacitance (Cm) changes | | | | |
| --- | --- | --- | --- | --- |
| N | **Treatment** | **ATP depletion (min)** | **Fold change** | ***p*-Value** |
| 10 | Ctrl | 6 | 1.22 ± 0.05 |  |
| 10 | Scram | 6 | 1.20 ± 0.02 |  |
| 10 | SLC | 6 | 1.05 ± 0.04 | <0.0109 vs. Ctrl, Scram |
| 10 | Ctrl | 7 | 1.27 ± 0.07 |  |
| 10 | Scram | 7 | 1.22 ± 0.02 |  |
| 10 | SLC | 7 | 1.03 ± 0.03 | <0.0053 vs. Ctrl, Scram |
| 10 | Ctrl | 8 | 1.26 ± 0.06 |  |
| 10 | Scram | 8 | 1.24 ± 0.02 |  |
| 10 | SLC | 8 | 1.09 ± 0.04 | <0.0080 vs. Ctrl, Scram |
| 10 | Ctrl | 9 | 1.27 ± 0.07 |  |
| 10 | Scram | 9 | 1.24 ± 0.03 |  |
| 10 | SLC | 9 | 1.07 ± 0.03 | <0.0051 vs. Ctrl, Scram |
| 10 | Ctrl | 10 | 1.28 ± 0.08 |  |
| 10 | Scram | 10 | 1.25 ± 0.03 |  |
| 10 | SLC | 10 | 1.07 ± 0.03 | <0.0023 vs. Ctrl, Scram |

Data are presented as mean ± S.E.M

**Table S16** Summary of SLC26A11 expression in rat brains after stroke (Fig. 6**b**)

| Summary of SLC26A11 expression level | | | | | | |  |
| --- | --- | --- | --- | --- | --- | --- | --- |
| Scram | | | | | **SLC** | |  |
| N | **% of baseline** | **Treatment** | **N** | **% of baseline** | | ***p*-Value vs. Scram** | |
| 4 | 37.43 ± 1.79 | Con | 4 | 33.79 ± 3.41 | | 0.380467 | |
| 4 | 55.20 ± 6.39 | Ipsi | 4 | 31.31 ± 2.84 | | 0.0141785 | |

Data are presented as mean ± S.E.M

**Table S17** Summary of infarct area (Fig. 6**d**)

| Summary of infarct area | | | |
| --- | --- | --- | --- |
| N | **Treatment** | **Infarct area (%)** | ***p*-Value** |
| 7 | Vehicle | 24.23 ± 2.52 |  |
| 9 | Scram | 20.97 ± 1.36 |  |
| 7 | SLC | 13.56 ± 2.61 | 0.0082, F (2, 20) = 6.158 vs. Vehicle, Scram |

Data are presented as mean ± S.E.M

**Table S18** Comparison of neurological severity scores (Fig. 6**e**)

| Comparison of neurological severity scores | | | | |
| --- | --- | --- | --- | --- |
| N | **Treatment** | **After stroke reperfusion (days)** | **Neurological severity scores** | ***p*-Value** |
| 6 | Vehicle | 3 | 2.67 ± 0.21 |  |
| 10 | Scram | 3 | 2.50 ± 0.17 |  |
| 11 | SLC | 3 | 1.91 ± 0.09 | < 0.0127 vs. Vehicle, Scram |
| 6 | Vehicle | 5 | 2.50 ± 0.22 |  |
| 10 | Scram | 5 | 2.20 ± 0.13 |  |
| 11 | SLC | 5 | 1.55 ± 0.16 | < 0.0050 vs. Vehicle, Scram |
| 6 | Vehicle | 7 | 2.17 ± 0.17 |  |
| 10 | Scram | 7 | 1.80 ± 0.13 |  |
| 11 | SLC | 7 | 1.27 ± 0.14 | < 0.0299 vs. Vehicle, Scram |

Data are presented as mean ± S.E.M

**Table S19** Assessment of motor functions by Rotarod test (Fig. 6**f**)

| Assessment of motor functions by Rotarod test | | | | |
| --- | --- | --- | --- | --- |
| N | **Treatment** | **After stroke reperfusion (days)** | **% of baseline** | ***p*-Value** |
| 6 | Vehicle | 1 | 35.45 ± 8.27 |  |
| 6 | Scram | 1 | 32.10 ± 6.38 |  |
| 7 | SLC | 1 | 62.11 ± 7.85 | < 0.0065 vs. Vehicle, Scram |
| 6 | Vehicle | 3 | 48.54 ± 2.40 |  |
| 6 | Scram | 3 | 51.95 ± 6.47 |  |
| 7 | SLC | 3 | 78.61 ± 5.10 | < 0.0065 vs. Vehicle, Scram |

Data are presented as mean ± S.E.M
