# Supplementary figures and images for: SLC26A11 Inhibition Reduces Oncotic Neuronal Death and Attenuates Stroke Reperfusion Injury
Source: Mol Neurobiol. 2023 Jun 28;60(10):5931–43. doi: 10.1007/s12035-023-03453-1 (PMC10471688; doi:10.1007/s12035-023-03453-1)

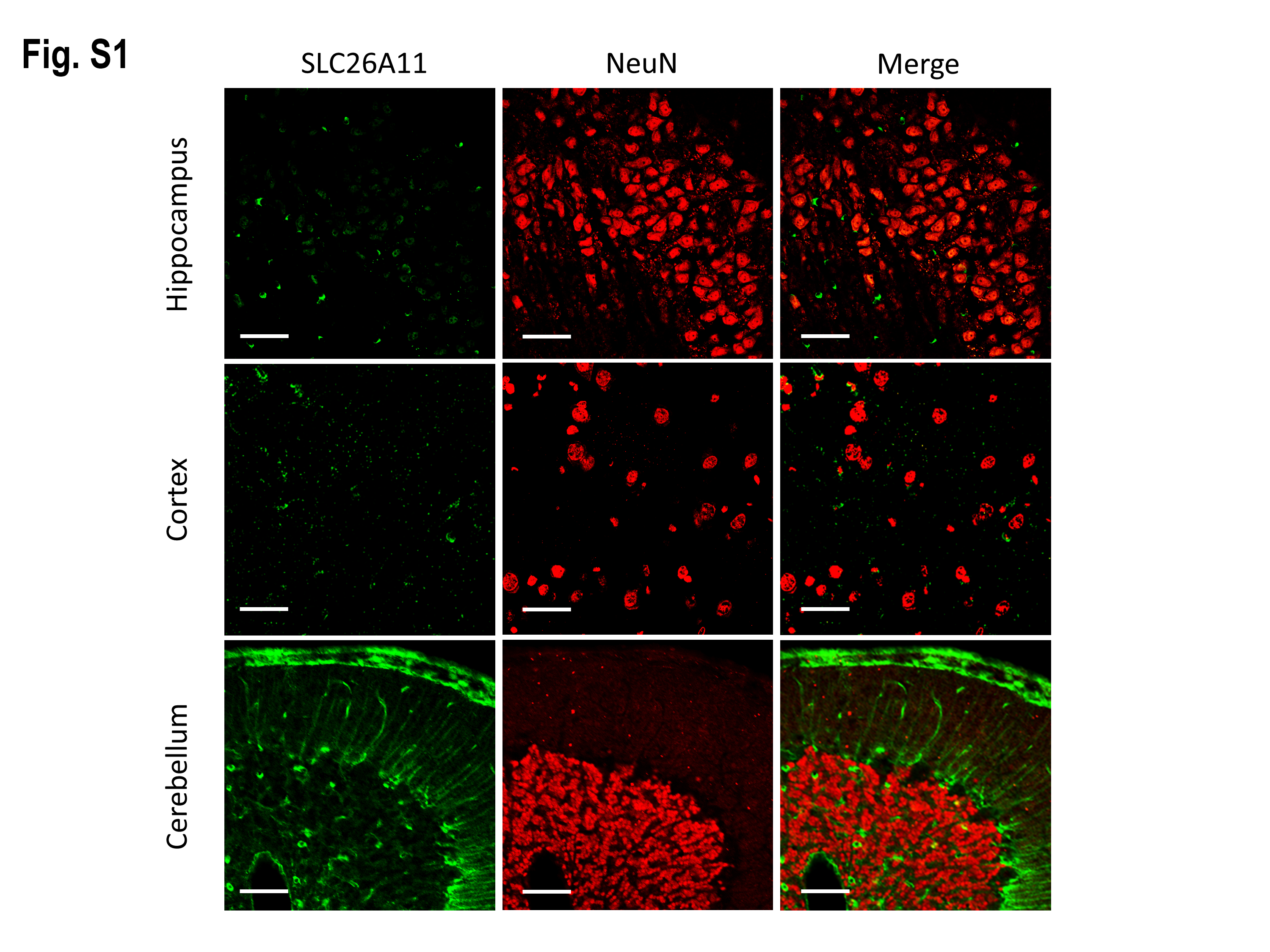

Supplement: Supplementary file 3 — High resolution image (TIF 3397 kb) [file 12035_2023_3453_MOESM2_ESM.tif]

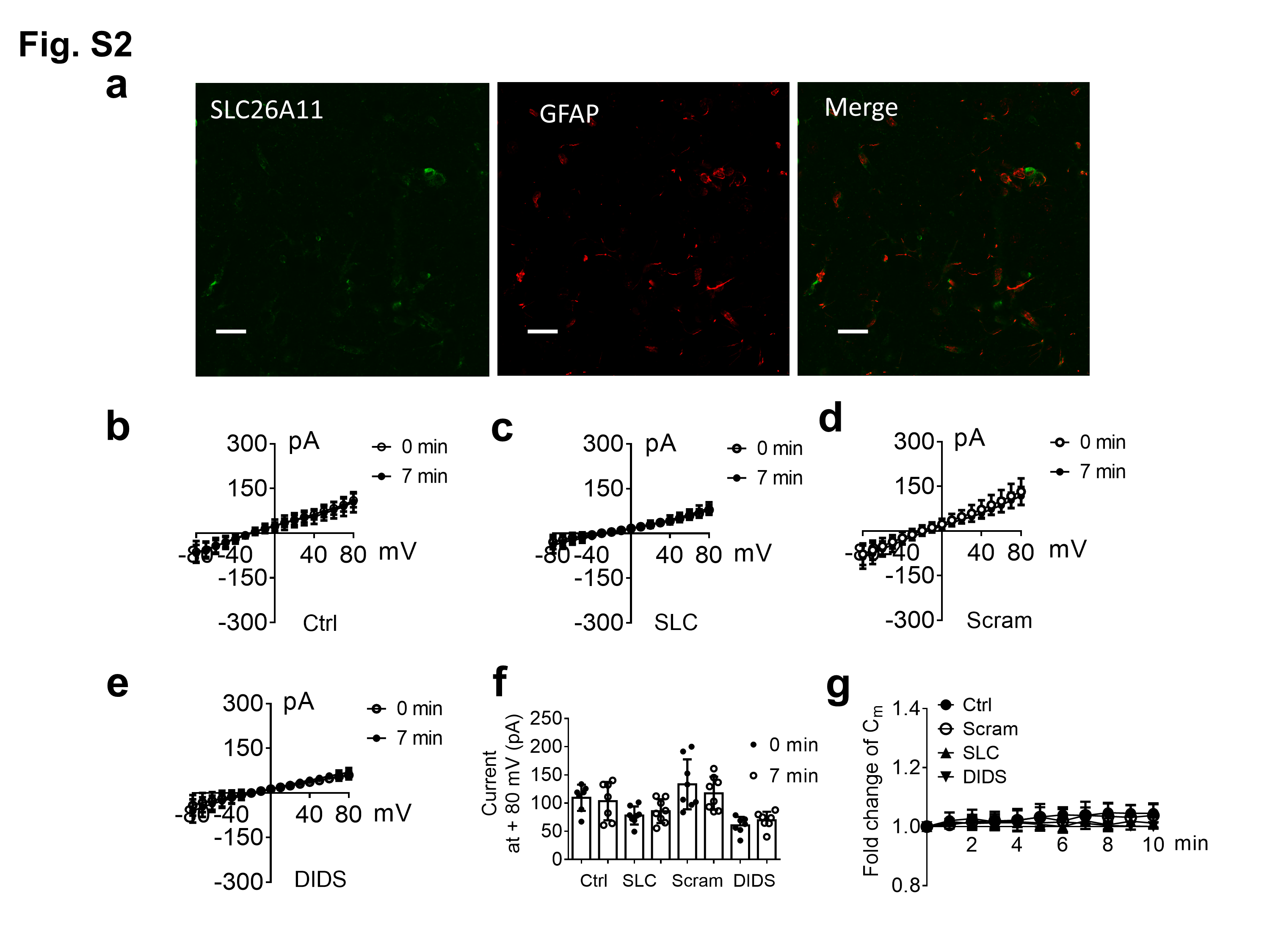

Supplement: Supplementary file 4 — (PNG 639 kb) [file 12035_2023_3453_Fig9_ESM.png]

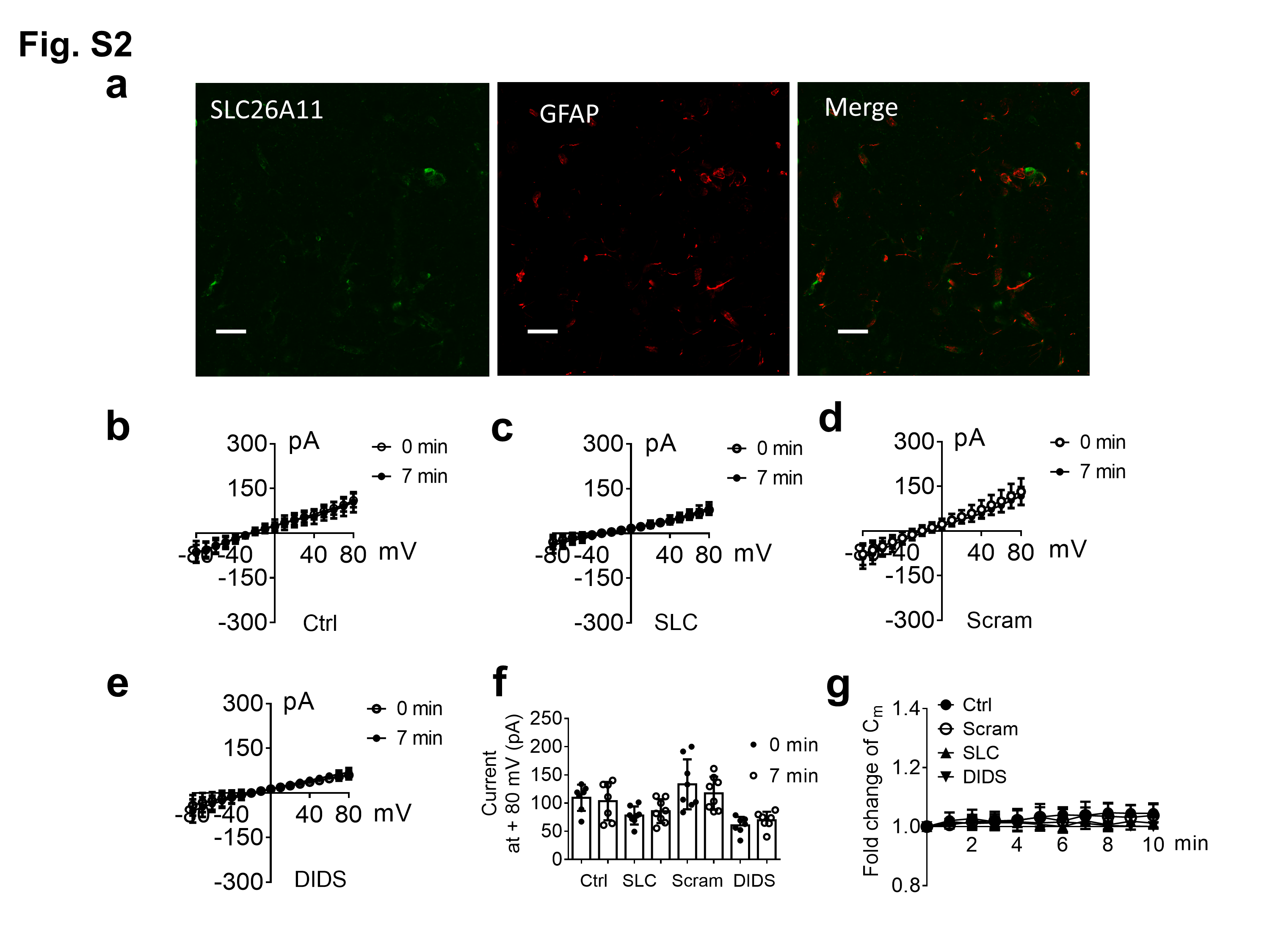

Supplement: Supplementary file 5 — High resolution image (TIF 1185 kb) [file 12035_2023_3453_MOESM3_ESM.tif]

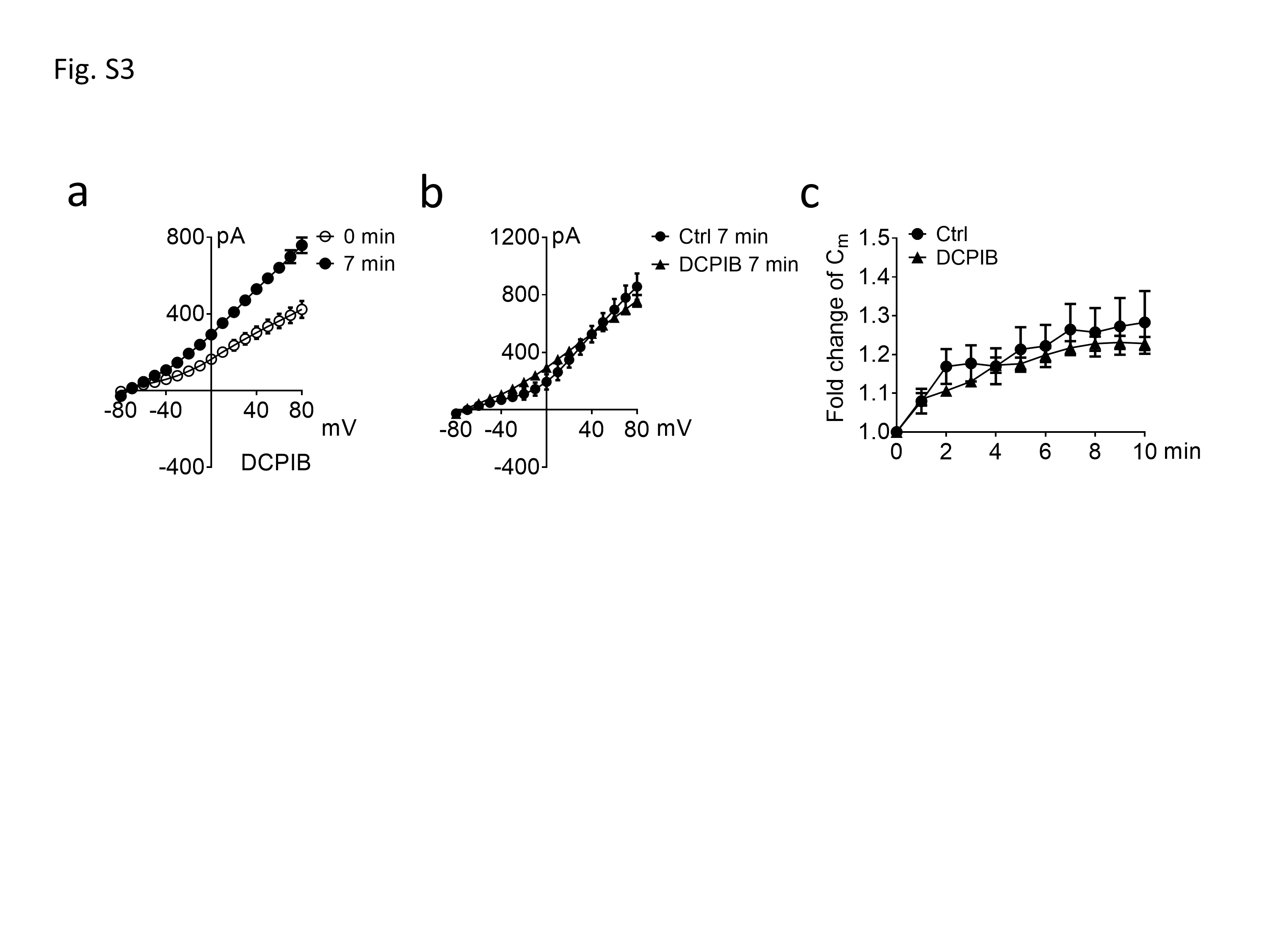

Supplement: Supplementary file 6 — (PNG 110 kb) [file 12035_2023_3453_Fig10_ESM.png]

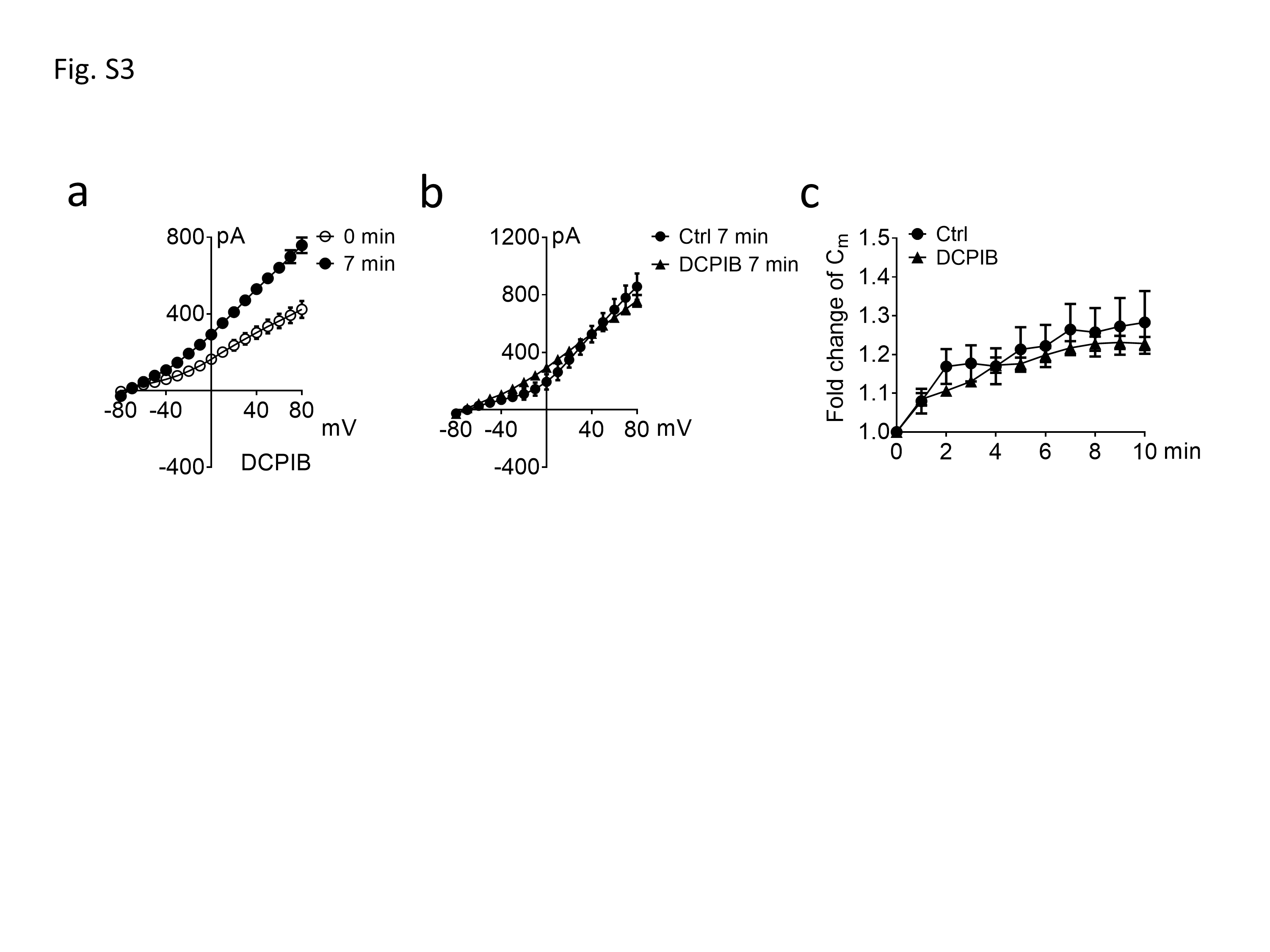

Supplement: Supplementary file 7 — High resolution image (TIF 490 kb) [file 12035_2023_3453_MOESM4_ESM.tif]
